# Role of gas–molecular cluster–aerosol dynamics in atmospheric new-particle formation

**DOI:** 10.1038/s41598-022-14525-y

**Published:** 2022-06-16

**Authors:** Tinja Olenius, Pontus Roldin

**Affiliations:** 1 Swedish Meteorological and Hydrological Institute, 60176 Norrköping, Sweden; 2 Division of Nuclear Physics, Department of Physics, Lund University, P. O. Box 118, 221 00 Lund, Sweden

**Keywords:** Atmospheric chemistry, Atmospheric chemistry, Macromolecules and clusters, Atmospheric science, Atmospheric chemistry

## Abstract

New-particle formation from vapors through molecular cluster formation is a central process affecting atmospheric aerosol and cloud condensation nuclei numbers, and a significant source of uncertainty in assessments of aerosol radiative forcing. While advances in experimental and computational methods provide improved assessments of particle formation rates from different species, the standard approach to implement these data in aerosol models rests on highly simplifying assumptions concerning gas–cluster–aerosol dynamics. To quantify the effects of the simplifications, we develop an open-source tool for explicitly simulating the dynamics of the complete particle size spectrum from vapor molecules and molecular clusters to larger aerosols for multi-compound new-particle formation. We demonstrate that the simplified treatment is a reasonable approximation for particle formation from weakly clustering chemical compounds, but results in overprediction of particle numbers and of the contribution of new-particle formation to cloud condensation nuclei for strongly clustering, low-concentration trace gases. The new explicit approach circumvents these issues, thus enabling robust model–measurement comparisons, improved assessment of the importance of different particle formation agents, and construction of optimal simplifications for large-scale models.

## Introduction

Aerosol particles are a key component of air pollution and Earth’s radiation budget, with aerosol–cloud interactions comprising a major uncertainty source in assessments of global radiative forcing^1,2^. Aerosols act as cloud condensation nuclei (CCN), which makes aerosol number concentrations a central factor affecting cloud properties and lifetime through effects on CCN concentrations^3^. As formation of secondary particles from condensable vapors makes a significant contribution to aerosol and CCN numbers^4,5^, adequate representation of new-particle formation in atmospheric models is critical for assessments of the climatic impacts of aerosols.

The effects of secondary particle formation on aerosol number size distributions and concentrations of CCN-relevant particle sizes are governed by complex aerosol dynamics processes, including initial clustering of gas-phase molecules, condensational growth of molecular clusters and larger aerosols, coagulation, and external particle sources and sinks^6,7^. The formation and growth rates critically depend on the ambient conditions and the chemical identities of the available clustering and condensing vapors, as the abilities of atmospheric gases to bind into stable clusters and/or condense on pre-existing surfaces of different compositions vary widely^8–11^. The initial formation rate *J* of new particles of ca. 1–2 nm is a key quantity that determines the upper limit for secondary particle concentrations, and vast efforts have been made to assess *J* for different chemical systems and environments in laboratory, field and modeling studies^12,13^.

*J* follows from molecular cluster distribution dynamics which are analogous to aerosol dynamics, with attachments and evaporations of vapor molecules to and from clusters, as well as cluster coagulation and removal^14,15^. The clustering process is not, however, explicitly included in standard atmospheric aerosol models that simulate the evolution of a particle size distribution. Instead, new particles are introduced at the smallest size covered by the model according to a given rate *J*, which is assumed to be unambiguously determined by the ambient conditions, including vapor concentrations, temperature, and possibly other parameters such as ion production rate^12^. This standard approach involves certain inherent assumptions on cluster kinetics and gas–cluster–aerosol interactions^16–18^: first, unlike the aerosol distribution, the cluster distribution which yields the formation rate *J* is assumed to be in a steady state corresponding to the ambient conditions. Second, small clusters are linked to the population of larger nanoparticles solely through *J*, and their formation and coagulation onto larger particles do not affect the concentrations of the clustering vapors or the aerosol size distribution. Such simplifications reduce model complexity and computational burden, and are necessary for large-scale models. However, their possible effects on modeled aerosol numbers in realistic atmospheric environments are not known.

In order to robustly assess and quantify the effects of the simplifications in gas–cluster–aerosol dynamics, explicit modeling of the time evolution and interactions among the whole nanoparticle size spectrum is needed. This is enabled by so-called discrete–sectional models, which treat the formation and growth of the smallest clusters molecule-by-molecule, and apply a sectional approach for larger particles^19^. However, discrete–sectional models are designed for chemically simple systems, and typically consider only one representative chemical compound. This is a very restrictive assumption for atmospheric aerosol formation, in which different compounds drive different stages of the formation and growth process^10,20,21^. Ideally, an atmospheric cluster–aerosol dynamics framework should be applicable to multiple, arbitrary compounds, given that chemical information is available. Such complex, flexible models are very challenging to construct and therefore have not existed to date.

In this work, we develop and apply an explicit multi-compound cluster dynamics model plugin that includes all kinetic processes between gas, cluster and aerosol phases and can be coupled to any aerosol dynamics model. Here, we implement the cluster plugin in an atmospheric trajectory model with a detailed aerosol size distribution representation. We perform simulations for a wide range of atmospheric conditions using different representative cluster formation chemistries, demonstrating that the omission of gas–cluster–aerosol dynamics and interactions can have significant effects on ultrafine particle and CCN number concentrations. This is the first study that comprehensively explores the effects of such processes on new-particle formation dynamics and aerosol size distributions. The open-source modeling tools introduced in this work can be applied in computationally light-weight box and local-scale models for an improved description of secondary aerosol formation. Such a set-up can be used to evaluate simplifications that are unavoidable for larger-scale models, and to assess the direction and magnitude of their effects for given atmospheric environments. This enables improved interpretation of large-scale model predictions, and opens possibilities for seeking optimal approaches to reduce the potential biases.

## Results

### Implementation of explicit gas–cluster–aerosol dynamics in an aerosol model framework

Standard atmospheric aerosol models implement initial particle formation by introducing new particles in the smallest included particle size bin, typically corresponding to a diameter of ca. 1–2 nm, according to a formation rate determined by the ambient conditions. This standard approach involves the following key assumptions and simplifications:

1. The molecular clusters below the smallest size bin are in a steady state corresponding to the ambient conditions, and their formation does not affect the vapor concentrations.
2. The cluster and aerosol distributions are linked solely through the formation rate of new particles of pre-determined size and composition. Cluster scavenging by larger nanoparticles does not affect the sizes of the particles, and potential aerosol particle shrinkage does not affect the cluster concentrations.

**Figure 1:**
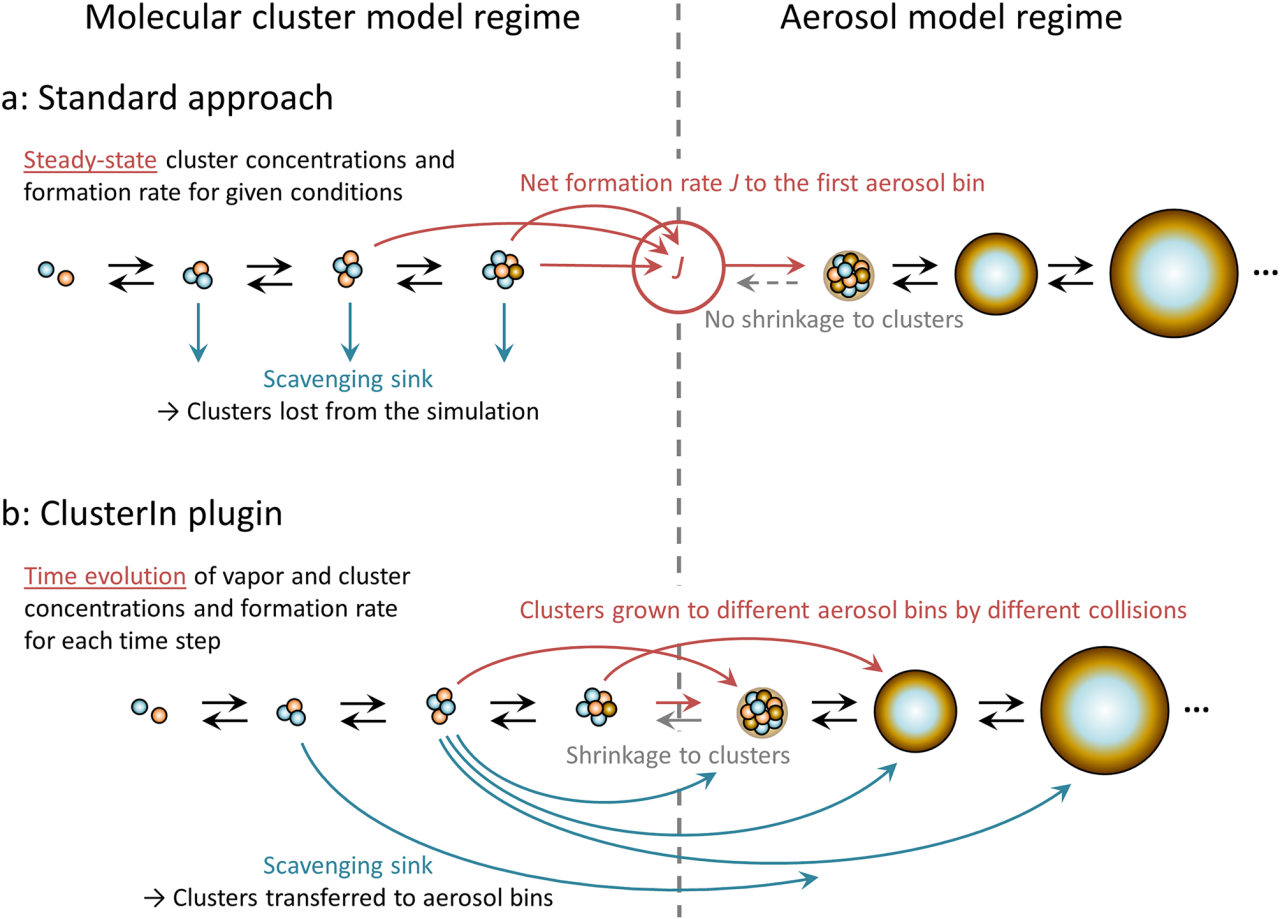
A schematic presentation of the treatment of gas–cluster–aerosol dynamics processes assuming the standard simplifications, and within an explicit framework. (**a)** The standard aerosol dynamics model set-up. (**b)** The present explicit approach, based on coupling a molecular cluster extension to an aerosol dynamics model.

In order to circumvent these restrictions, we develop an explicit approach based on a direct coupling of cluster and aerosol distribution dynamics models. The coupling is implemented through a cluster model plugin that can be embedded in any aerosol model framework as described in “Methods” and Supplementary Information. The cluster dynamics plugin, hereafter abbreviated as ClusterIn, serves as an extension of the aerosol model down to molecular cluster sizes, thus enabling the simulation of the time evolution of the complete cluster and particle size range. The treatment of the gas–cluster–aerosol dynamics processes in the standard and present approaches is depicted by Fig. 1 and summarized in Table 1. Table 1 also lists effects that the standard simplifications can be expected to have, and ambient conditions in which the effects may be particularly important.

**Table 1:** Treatment of different molecular cluster dynamics processes in the standard approach and in the explicit approach developed in this work. The listed potential effects of the standard simplifications are general examples, and the effects can be more diverse over various atmospheric conditions.

Dynamic process	Standard approach	Explicit approach	Potential effects of standard simplifications	Environments and cluster chemistries that are expected to be especially affected
Time evolution of cluster concentrations	Clusters are assumed to be in instantaneous steady state at every model time step	The time evolution is explicitly simulated	Dependent on the time evolution of ambient conditions; overprediction of formation rate likely at increasing vapor concentrations, underprediction at decreasing concentrations^18^	Conditions with long time scales of cluster formation, such as low vapor concentrations
Effect of cluster formation on vapor concentrations	Clustering does not affect vapor concentrations (vapors may be reduced according to the particle formation rate, but the molecules bound in cluster phase are omitted)	Time evolution of vapor concentrations during clustering is explicitly simulated	Overprediction of vapor concentrations and thereby particle formation rate	Strongly clustering compounds, low vapor concentrations
Sizes and compositions of newly-formed particles	All new particles formed by a given chemical mechanism are assumed to have the same molecular composition	All new particles growing out of the cluster regime through different cluster–molecule or cluster–cluster collisions are placed in the aerosol size bin that corresponds to the size of the collision product	Underprediction of sizes of new particles; inaccuracies in composition	Chemistries involving cluster–cluster coagulation, multi-component clustering including molecules and clusters of different sizes
Cluster scavenging by aerosol particles	Effect of a given sink on formation rate can be included, but scavenged clusters are lost from the cluster–aerosol system^a^ (Scavenging is omitted or the sink is independent of aerosol concentrations)^b^	Cluster–aerosol collisions are included and the collision products are distributed to the aerosol bins	Underprediction of aerosol growth due to omission of scavenged clusters^a^ (Inaccuracies in formation rate)^b^	Strongly clustering compounds for which a significant amount of vapor is bound in cluster phase^a^ (Environments with significant and/or varying sinks)^b^
Aerosol evaporation beyond the smallest size covered by the aerosol model	Shrunken aerosols are removed from the particle spectrum, and the material is either lost or transferred to gas phase	Evaporated aerosols are transferred to the cluster regime	Underprediction of cluster concentrations and formation rate	Decreasing vapor concentrations with small particles that contain volatile compounds

^a^Applied here (see “Methods”).

^b^The simplest approach for cluster scavenging, relevant to simplified theories (e.g. classical nucleation theory) or experimental formation rates determined at constant sink.

To test the effects of the standard simplifications in realistic atmospheric conditions, we apply ClusterIn in the trajectory model ADCHEM, which is a Lagrangian chemical transport model with a detailed aerosol dynamics description^22,23^. ADCHEM is operated along several trajectories around a Northern European domain as shown in Supplementary Fig. S3. The set-up has been previously benchmarked against ambient aerosol and trace gas measurements^23^, and the trajectories were selected to cover various ambient conditions from remote to more polluted environments (Supplementary Table S2 and Fig. S4). Simulations were performed applying either the standard assumptions, or the explicit gas–cluster–aerosol dynamics as described in Table 1 and “Methods”.

Cluster formation is assumed to occur through either sulfuric acid (H_2_SO_4_) and ammonia (NH_3_), or sulfuric acid and dimethylamine (DMA), which represent weaker and stronger clustering chemistries, respectively. These chemistries are applied as they have a central role in atmospheric particle formation^9,20,24,25^, and the underlying clustering mechanisms are well understood^8,26^. A weakly clustering chemical system (here H_2_SO_4_–NH_3_) forms molecular clusters that evaporate significantly, and therefore requires sufficiently high vapor concentrations for particle formation. Clusters in a strongly clustering system (here H_2_SO_4_–DMA) have low evaporation rates, resulting in particle formation also at low vapor concentrations. Using representative, well-characterized compounds enables a reliable assessment of the potential artefacts that the conventional simplifications may cause for different types of cluster formation chemistries and environments. Other compounds can be straight-forwardly applied in the ClusterIn framework as the detailed chemical information becomes available.

**Figure 2:**
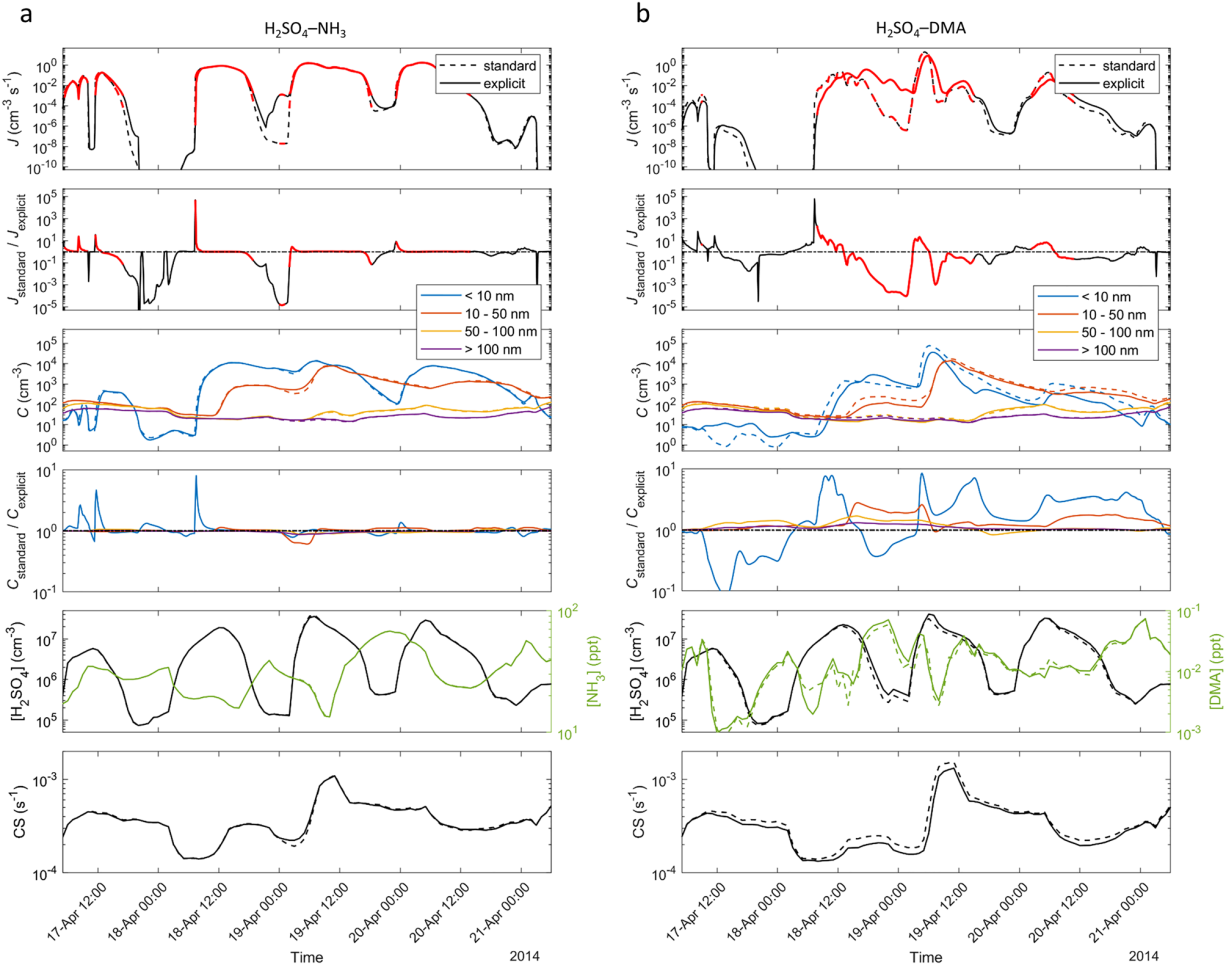
Particle formation rate (*J*), size-classified particle concentrations (*C*), concentrations of clustering vapors ([H_2_SO_4_] and [NH_3_] or [DMA]) and H_2_SO_4_ condensation sink (CS) along trajectory 7 (UTC time) for the standard and explicit simulation cases with initial particle formation from H_2_SO_4_–NH_3_ (**a**), and H_2_SO_4_–DMA (**b**). For *J* and *C*, also the ratio between the standard and explicit cases is shown, with the horizontal black dash-dotted line depicting a ratio of one. Periods during which *J* ≥ 10^−3^ cm^−3^ s^−1^ for at least one of *J*_standard_ and *J*_explicit_ are marked with red in the time series of *J*. Note that both NH_3_ and DMA are present in all simulations, and the difference between the H_2_SO_4_–NH_3_ and H_2_SO_4_–DMA cases is only the chemical mechanism assumed for the initial particle formation.

Figure 2 shows an example of the particle formation rate *J* and the particle number concentrations *C* along a single trajectory for the standard and explicit cases with initial particle formation from either H_2_SO_4_–NH_3_ or H_2_SO_4_–DMA. The trajectory includes transport over relatively clean environments in Scandinavia, Baltic Sea and Finland. To distinguish particle size regimes to which the formation dynamics effects may extend, particle concentrations are size-classified to four size ranges corresponding to small nucleation-mode particles (particle diameter *d*_p_ < 10 nm), lower and upper Aitken-mode particles (*d*_p_ = 10–50 nm and 50–100 nm, respectively), and sizes larger than the ultrafine range (*d*_p_ > 100 nm).

For these conditions, the standard assumptions cause visible changes in *J* and *C* that are minor in the case of particle formation from H_2_SO_4_–NH_3_ and more distinct for H_2_SO_4_–DMA. While both under- and overprediction of *J* and *C* occur along the trajectory, some trends can be observed: first, the bias in *J*_standard_ often alters according to the time evolution of the precursor vapors, here mainly H_2_SO_4_. Especially for the H_2_SO_4_–NH_3_ case, overprediction tends to occur at increasing [H_2_SO_4_], followed by less biased values during the period of maximum [H_2_SO_4_] and underprediction at decreasing [H_2_SO_4_]. This is due to the steady-state assumption, which causes *J*_standard_ to reflect changes in the ambient conditions with no delay, and may also lead to overpredicted maxima and underpredicted minima. Second, the differences between the standard and explicit cases are larger and more variable for H_2_SO_4_–DMA, with non-negligible effects on also the precursor vapor concentrations and condensation sink (CS). Particle formation pathways driven by such strongly-binding, low-volatile vapors are likely to exhibit stronger feedbacks between vapor concentrations, formation rate and particle numbers since cluster formation and vapor condensation onto larger nanoparticles can be significant sinks of vapor, resulting in stronger dynamic couplings. Yet, the effects may be different in different types of environments.

### Effects on aerosol number concentrations

**Figure 3:**
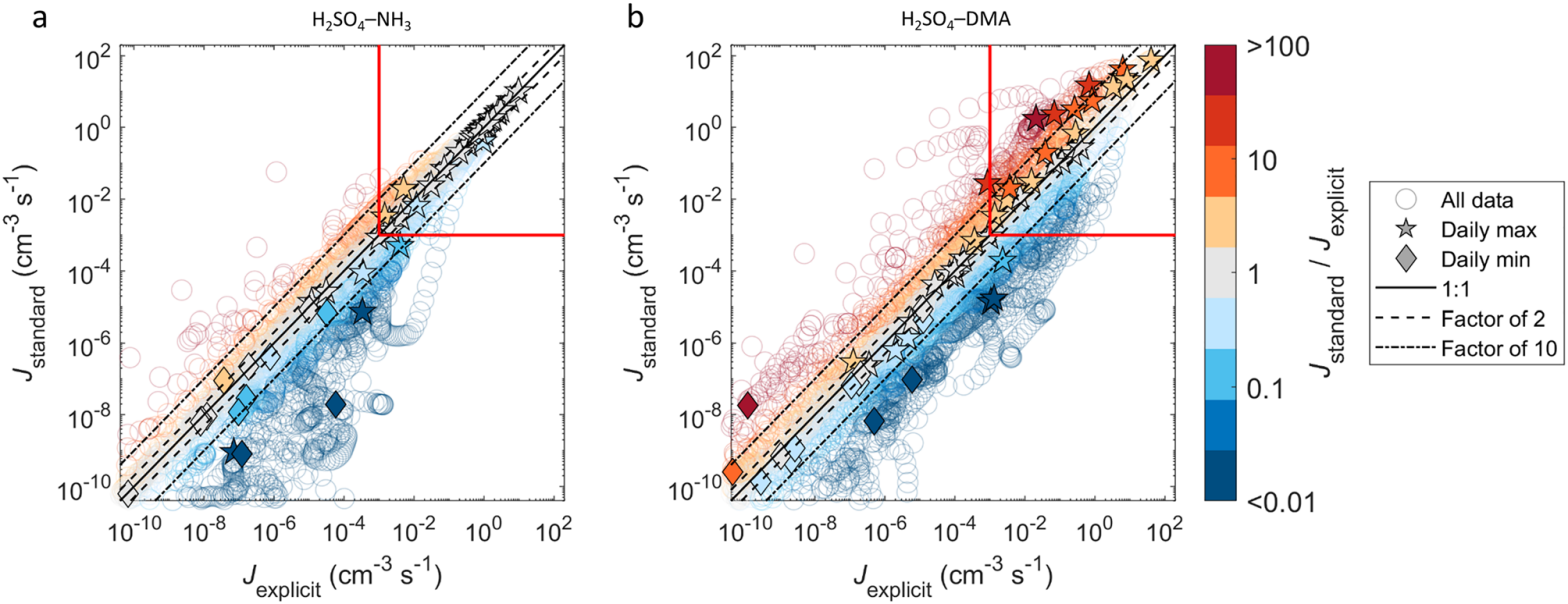
Particle formation rates for the standard and explicit cases for all trajectories with the formation process driven by H_2_SO_4_–NH_3_ (**a**), and H_2_SO_4_–DMA (**b**). The color scale gives the ratio between the standard and explicit cases. The lower limits of the axes are set to 10^−10^ cm^−3^ s^−1^ to exclude rates that are close to zero, and the red rectangles mark the region of atmospherically significant formation rates of ≥ 10^−3^ cm^−3^ s^−1^.

**Figure 4:**
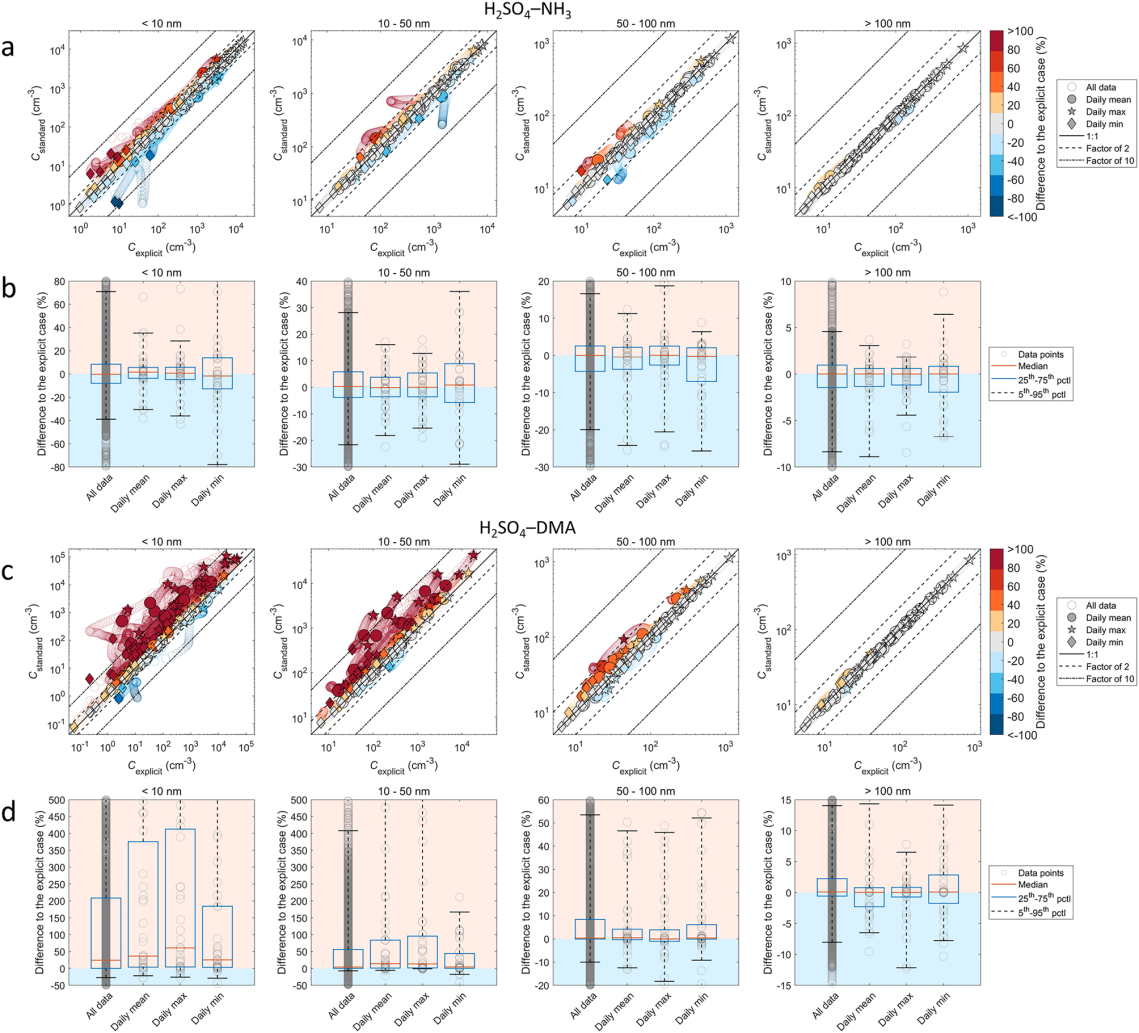
Size-classified particle number concentrations for the standard and explicit cases for all trajectories with initial particle formation from H_2_SO_4_–NH_3_ (**a,b**), and H_2_SO_4_–DMA (**c,d**). (**a,c)** show the absolute concentrations, and the color scale gives the difference Δ*C* = (*C*_standard_ − *C*_explicit_) / *C*_explicit_ between the standard and explicit cases. (**b,d)** show statistics for the difference Δ*C*, with the corresponding data points Δ*C* shown in light grey. In (**b,d)**, regimes of under- and overprediction are depicted by light blue and light red shades, respectively. Scales of y-axes are limited to ranges covering the 5th–95th percentiles of Δ*C*, except for the smallest sizes in (**b)** for which the 95th percentiles extend to up to ca. 190% for < 10 nm (corresponding to ca. threefold overprediction), and (**d)** for which the extent is up to ca. 5000% for < 10 nm (ca. 50-fold overprediction) and up to ca. 1000% for 10–50 nm (ca. tenfold overprediction).

To assess the overall effects of the simplifications and the significance of the gas–cluster–aerosol dynamics processes over various atmospheric conditions, the results of all trajectories are collected in Figs. 3 and 4. Figure 3 shows the formation rates *J*_standard_ vs. *J*_explicit_ for all individual data points in the simulated time series, as well as the daily maximum and minimum values. The daily extrema are included to distinguish potential time shift effects as, for example, the steady-state assumption tends to cause a time lag in the formation rate, but may not necessarily have a major effect on the magnitude of the maxima or minima (Fig. 2). In the case of H_2_SO_4_–NH_3_, *J*_standard_ differs from *J*_explicit_ at low formation rates (mainly at *J* ≲ 0.1 cm^−3^ s^−1^) and converges to *J*_explicit_ at higher rates. By contrast, applying particle formation from H_2_SO_4_–DMA results in differences between *J*_standard_ and *J*_explicit_ throughout the range of formation rates, with the standard approach overpredicting both the time-dependent *J* and the daily maximum *J* by factors of up to > 10–100 especially at higher formation rates (*J* ≳ 0.1–1 cm^−3^ s^−1^).

Effects on particle numbers are presented in Fig. 4. For each particle formation chemistry, the upper panel (a, c) shows the absolute particle numbers *C*_standard_ and *C*_explicit_, and the lower panel (b, d) shows the median and the 25th–75th and 5th–95th percentiles of the relative difference Δ*C* = (*C*_standard_ − *C*_explicit_)/*C*_explicit_. The upper panels indicate at which absolute concentrations effects of different magnitudes occur, while the lower panels provide an overview of the statistics of Δ*C* over all concentrations.

The effects are generally largest at the smallest particle sizes of ≲ 50 nm. For H_2_SO_4_–NH_3_, the differences between *C*_standard_ and *C*_explicit_ are mostly within a factor of ca. 2 and include both under- and overprediction of *C*. For H_2_SO_4_–DMA, however, the standard approach is biased towards overprediction of particle numbers by factors of up to ~ 10–100 at *d*_p_ < 10 nm and up to ~ 10 at *d*_p_ = 10–50 nm. The differences are more prominent for H_2_SO_4_–DMA also for sizes larger than 50 nm, at which they are generally within a factor ca. 2 for both chemistries. The largest sizes of *d*_p_ > 100 nm show differences of up to ca. ± 5–15% that converge to zero at higher absolute particle numbers at which the contribution of new-particle formation becomes minor compared to primary particle sources.

While differences in particle numbers are rather straight-forwardly linked to differences in *J* for the smallest particle sizes, it must be borne in mind that the effects—especially on larger sizes—also depend on ambient conditions. These include the absolute concentrations of particles and condensable vapors that largely determine particle survival probabilities and growth rates. Increase in the number of newly-formed particles may lead to either increase or decrease in the number of larger particles, the latter possibly resulting from slower growth due to less condensable vapor available per particle. Also feedbacks between formation rate and particle numbers may occur due to particles acting as a sink for vapors and clusters. The qualitative trends and the magnitude of the differences shown in Fig. 4 are thus more relevant than the exact quantitative values. To ensure that the trends are robust, we conducted several test simulations by varying the key input related to cluster formation and ambient conditions (Supplementary Information Sect. 4.1). The varied settings include the quantum chemical data set used for cluster evaporation rates, DMA emissions, and initial gas and particle concentrations for the trajectories. We also applied three-component cluster formation using previous data available for H_2_SO_4_–NH_3_–DMA, which is generally a less studied system. All test simulations give a similar result: in the presence of the strongly clustering, low-concentration DMA compound, the standard approach causes overprediction of particle numbers by up to ~ 1 to 2 orders of magnitude at sub-50 nm sizes, with the largest effects at sizes below 10 nm.

### Effects of different cluster dynamics processes

To determine the significance of each gas–cluster–aerosol dynamics process (Table 1), their effects are assessed as follows (see also Supplementary Information Sect. 4.2): first, to distinguish the effect of time-dependent cluster dynamics, the standard case was run without the steady-state assumption, also allowing vapor concentrations to evolve within ClusterIn (test 1). Test 1 is compared with the standard case, and the purpose of the test is to remove only the steady-state assumption with no other changes. Second, to assess the importance of different cluster–aerosol interactions, the explicit case was run with one of the following couplings removed: consideration of the exact sizes and compositions of clusters grown to aerosol regime (test 2), transfer of scavenged clusters to aerosol bins (test 3), and transfer of shrunken aerosols to cluster regime (test 4). Tests 2–4 are compared with the explicit case. Here, the purpose is to assess the magnitude of the error due to omitting one of the interactions. Note that due to the interlinked dynamic processes and feedbacks, the errors are not additive, that is, their sum does not equal the difference between the standard and the explicit cases (Fig. 4).

**Figure 5:**
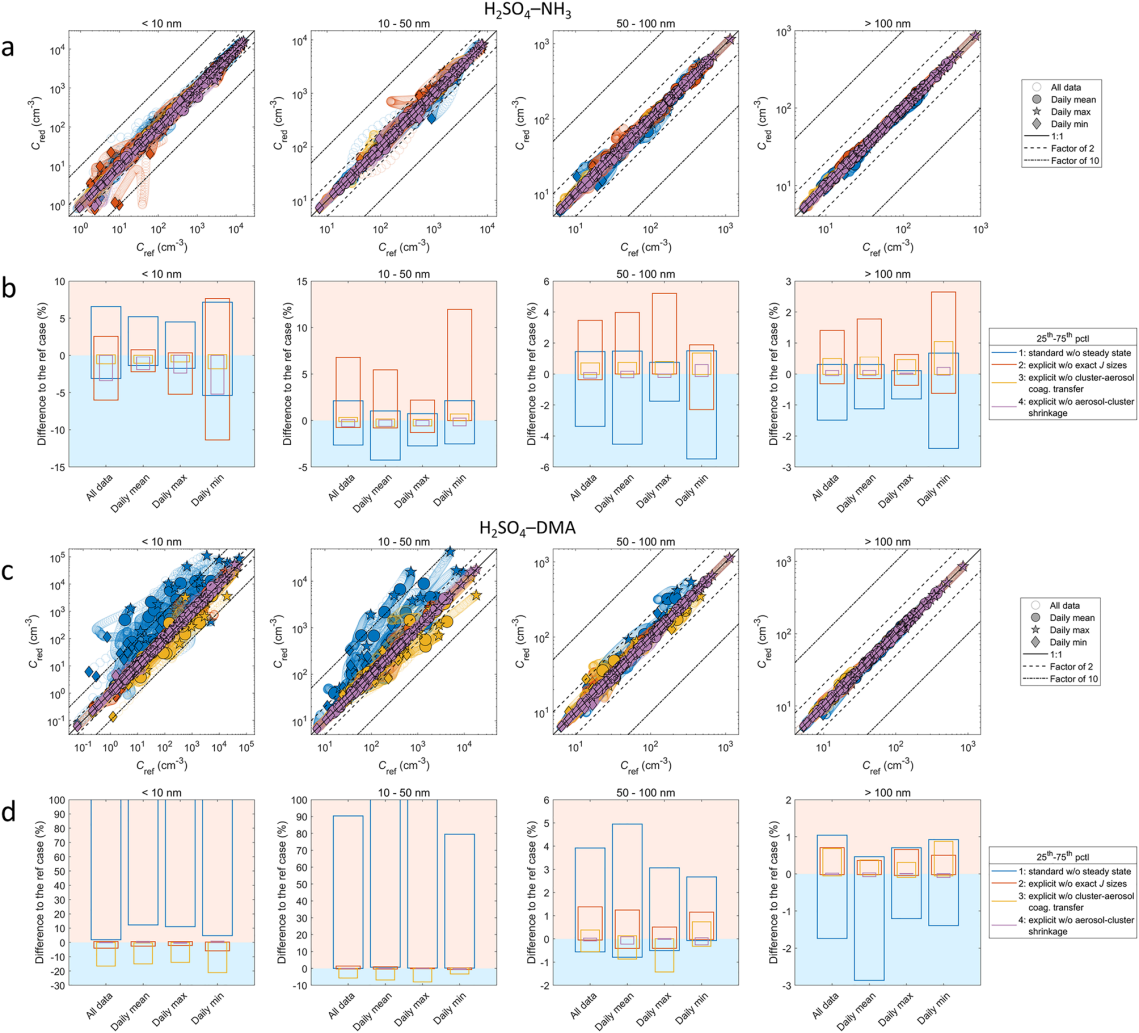
Particle number concentrations *C*_red_ and *C*_ref_ and their differences Δ*C*_test_ = (*C*_red_ − *C*_ref_) / *C*_ref_ for test simulations assessing the roles of different gas–cluster–aerosol dynamics processes for all trajectories with initial particle formation from H_2_SO_4_–NH_3_ (**a,b**), and H_2_SO_4_–DMA (**c**,**d**). Each test case “red” is compared to a reference case “ref”, where “red” refers to the reduced or simplified case, and “ref” to the benchmark case; that is, Δ*C*_test_ always corresponds to the error made due to the omission of one of the cluster dynamics processes (Supplementary Table S3). Each test is depicted by one color as shown in the legend. For figure clarity, only the 25th–75th percentiles of Δ*C*_test_ are shown in (**b,d)**. Scales of y-axes are limited to better show lower values in (**d)**, for which the 75th percentiles extend to up to several hundred percent for < 10 nm and up to ca. 150% for 10–50 nm.

The comparisons are shown in Fig. 5. For H_2_SO_4_–NH_3_, none of the individual tests show an exceptionally large effect that would dominate over all other effects. For H_2_SO_4_–DMA, by contrast, the steady-state assumption (test 1) has an overwhelming effect that leads to the overprediction of ultrafine particle numbers. While the steady-state assumption is generally likely to overpredict *J* e.g. at increasing vapor concentrations, the significant bias that it causes for H_2_SO_4_–DMA is linked to strong cluster formation and clusters acting as a sink for vapor, and DMA sources and concentrations typically being much lower than those of NH_3_. The low concentration can increase the magnitude of the steady-state effects, as it corresponds to longer time scales of cluster formation through molecular collisions. The net positive bias largely stems from the omission of vapor dynamics and vapor-to-cluster sink, which results in higher [DMA] and thereby in increased *J* (Supplementary Figures S9 and S10). The results suggest that such non-steady-state effects comprise the most significant errors that the simplified standard approach may involve.

Furthermore, omitting dynamic phenomena that tend to increase the survival of initial particles and/or *J* (tests 2–4) can be expected to decrease the number of small particles, given that possible feedbacks are not considered. In tests 2–4, deviations in particle numbers are mainly up to a factor of 2 at < 10 nm and mostly below it at larger sizes. An exception is the more prominent effect of aerosol growth by cluster–aerosol coagulation (test 3) for H_2_SO_4_–DMA, for which cluster concentrations are elevated due to the strong cluster binding. The initial particle size and the early growth at sizes down to ca. 1–1.5 nm are generally important for particle survival, as the scavenging rate decreases rapidly with particle size in this regime. Their effects are likely to be notable especially when using a high size resolution for the aerosol bins, such as in the present ADCHEM set-up.

We note that the dynamic effects can be different at highly polluted conditions such as megacities^25^. Specifically, high amine sources are expected to improve the performance of the steady-state approximation. We therefore tested hypothetical scenarios with increased anthropogenic emissions, which indeed lead to somewhat less overprediction (Supplementary Information Sect. 4.1; Fig. S7). However, low [H_2_SO_4_] and reduction of [H_2_SO_4_] during strong clustering can contribute to overprediction also at high [DMA] (Supplementary Fig. S8).

### Effects on cloud condensation nuclei number concentrations

**Figure 6:**
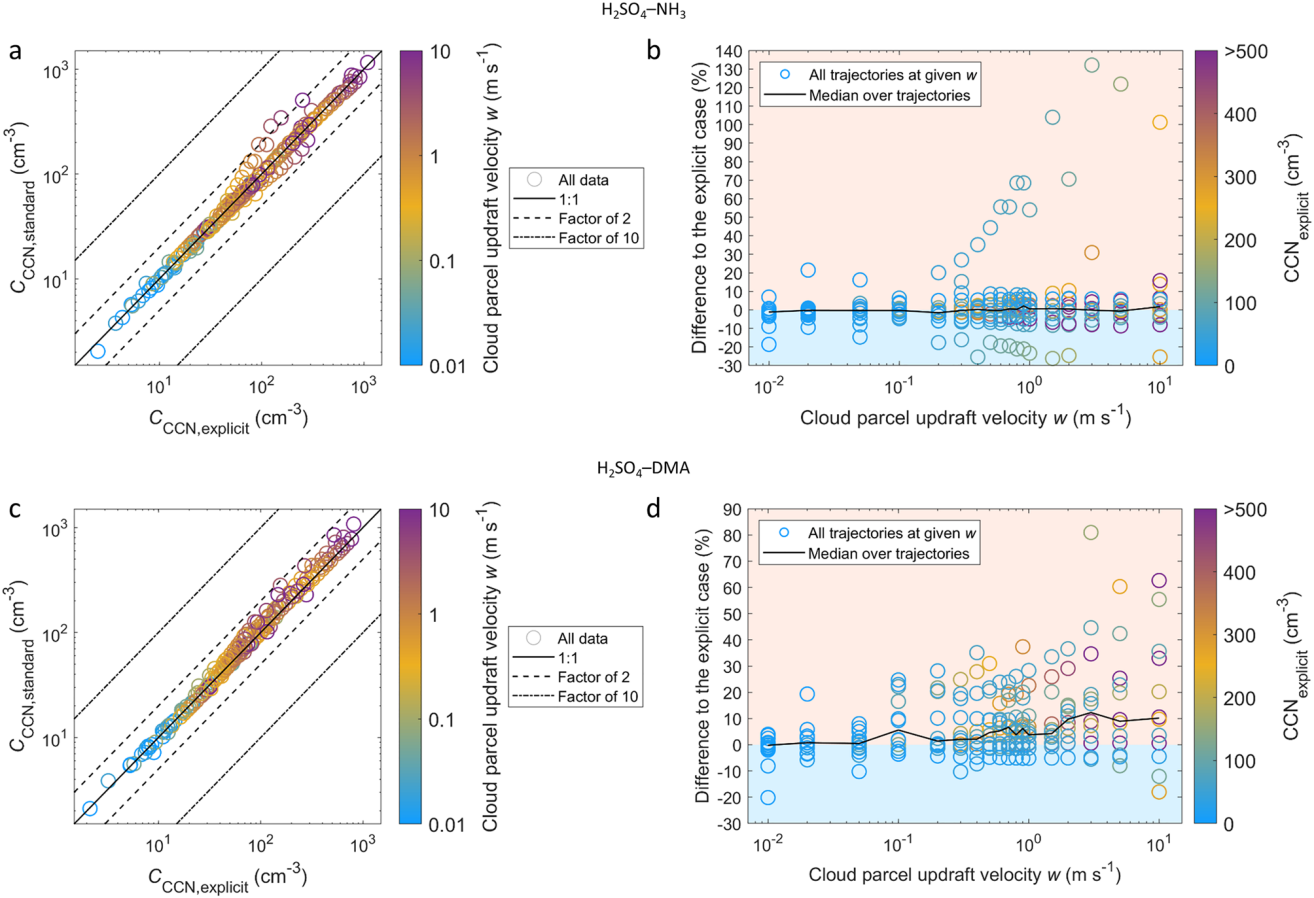
CCN number concentrations for the standard and explicit cases at the ends of the trajectories with initial particle formation from H_2_SO_4_–NH_3_ (**a,b**), and H_2_SO_4_–DMA (**c,d**). (**a,c)** show the absolute concentrations *C*_CCN_ at different cloud parcel updraft velocities *w*, as indicated by the color scale. (**b,d)** show the difference Δ*C*_CCN_ = (*C*_CCN,standard_ − *C*_CCN,explicit_) / *C*_CCN,explicit_ between the standard and explicit cases as a function of *w* for all trajectories, and the median of Δ*C*_CCN_ over the trajectories at given *w*. Regimes of under- and overprediction are depicted by light blue and light red shades, respectively.

In order to assess the effects on CCN number concentrations, we applied a cloud parcel model^27^ that simulates the activation of aerosol particles to CCN (“Methods”). CCN concentrations are modeled at the end of each trajectory, where the new particles formed along the trajectory have had the maximum time to grow to CCN-relevant sizes. Figure 6 shows the CCN concentrations *C*_CCN_ and the differences Δ*C*_CCN_ between the standard and explicit cases for different cloud parcel updraft velocities *w*. The effects on CCN are in line with the effects on particle numbers: the standard assumptions cause larger errors and a tendency towards overprediction of CCN numbers for H_2_SO_4_–DMA particle formation. It can be noted that for H_2_SO_4_–NH_3_, the largest effects (positive bias above ca. 35% in panel b) correspond solely to trajectory 12, which exhibits the largest differences in the concentrations of > 10 nm particles at the trajectory end. On the contrary, the positive bias for H_2_SO_4_–DMA (panel d) results from a consistent net effect over all trajectories at given *w*. The bias increases with increasing *w*, corresponding to smaller activation diameters and thereby higher *C*_CCN_. These results indicate that while the effects of the simplifications are most significant for the smallest particle sizes, they extend also to CCN-relevant sizes and therefore to predictions of aerosol–cloud interactions.

## Discussion

Advances in the development of instruments and computational tools have opened possibilities to explore the atmospheric molecular cluster phase^26^, which is the key for predicting the numbers and climatic effects of secondary aerosol particles. Here we present the next step in model development to probe new-particle formation dynamics by constructing an explicit plugin to simulate the time evolution and interactions of the entire gas–cluster–aerosol system. To our knowledge, this is the first study to assess the role of the cluster dynamics processes in realistic atmospheric environments, and to resolve the potential effects of reducing the representation of the cluster size regime to a simple steady-state formation rate. The trajectory simulation results demonstrate that the simplified standard approach is a reasonable approximation for environments where initial particle formation is driven by relatively weakly clustering compounds, here represented by H_2_SO_4_–NH_3_, which require higher vapor concentrations to yield non-negligible particle formation rates. Here, the effects of the simplifications on particle numbers are mostly within a factor of two. However, for particle formation from strongly clustering compounds such as H_2_SO_4_–DMA, that typically have low sources and concentrations, the simplifications lead to overprediction of ultrafine particle numbers, here by factors of up to ca. 10 for the lower Aitken mode (≲ 50 nm) and well beyond for the smallest sub-10 nm sizes. Biases of this order distort both model predictions and model validation against measurements. They also propagate to predictions of CCN numbers, causing an overprediction tendency in the contribution of particle formation to CCN.

These effects arise from the dynamics of a gas–cluster–aerosol system, and are thus not related to only specific chemical compounds. Similar chemistries are expected to behave similarly: for weakly clustering compounds, the concentration of clusters is typically negligible compared to the concentrations of the cluster-forming vapors. Such compounds may exhibit non-steady-state effects in cluster concentrations and formation rate during changes in vapor concentrations or other ambient conditions, but vapor concentrations can be expected to remain constant during clustering. Examples of weak clustering agents are weakly-binding acid–base mixtures such as H_2_SO_4_–NH_3_^28^, other acidic species such as iodic acid^29^, and oxidized organic compounds^30^.

Strongly clustering compounds, on the other hand, are not as abundant in the atmosphere due to low sources, dilution and removal by condensation. Their low concentrations and efficient clustering can lead to stronger dynamic feedbacks between the vapors and clusters, and a significant fraction of vapors being taken up by the clusters. This makes the steady-state assumption substantially worse and biased towards overprediction. This type of compounds include acid–base mixtures with low trace concentrations of strong base compounds, for example DMA and other strong monoamines^31^, diamines^32^ and guanidine^33^. This applies also to multi-compound clustering pathways that are significantly affected by a strong clustering agent, as demonstrated here for the three-component H_2_SO_4_–NH_3_–DMA chemistry, and to a combination of low H_2_SO_4_ and high amine concentrations. The latter can occur in polluted megacity environments especially when sulfur emissions are subject to more stringent abatement strategies than base emissions^34^. Finally, such effects may also be relevant in the upper troposphere, where vapor concentrations are generally lower than in the boundary layer and clustering is enhanced by low temperatures^35^.

In addition to the steady-state approximation, dynamic interactions between cluster and aerosol distributions may affect particle concentrations through effects on particle survival, which depends on initial particle sizes and growth. This is relevant for detailed studies of aerosol formation and growth mechanisms. The present results indicate non-negligible contribution of cluster–aerosol coagulation to nanoparticle growth for the H_2_SO_4_–DMA case. Omitting this effect may lead to decreased particle numbers due to decreased survival.

The model results complement experimental observations of cluster formation efficiencies for atmospheric chemical compounds. Laboratory experiments of new-particle formation are generally designed for determining formation rates at unambiguous, well-defined steady-state conditions at constant vapor sources. Models benchmarked against experiments can subsequently be applied to assess the formation dynamics in atmospheric environments involving time-dependent vapor and particle sources and sinks. Here, we use representative cluster chemistries and quantum chemical methods that have been previously validated against steady-state measurements, showing an agreement with the trends, magnitude and often even quantitative values of observed formation rates^8,36,37^. The efficient clustering of DMA and other amines has been demonstrated in various measurements^8,31,38^, in line with model predictions. Specifically, strong clustering has been shown to result in reduction of vapor and elevated cluster concentrations for a H_2_SO_4_–DMA mixture^39^. The experimentally observed enhancement in nanoparticle growth and survival has been attributed to coagulation by chemically simplified cluster–aerosol dynamics models^9,39^, as also indicated by our results. This work sets such observations to the context of dynamic atmospheric conditions by demonstrating the implications for ambient aerosol and CCN numbers.

To summarize, we provide a tool for explicit description of gas–cluster–aerosol couplings and nanoparticle size distribution dynamics that is needed for (1) detailed understanding of particle formation mechanisms and model–measurement comparisons, and (2) deriving robust simplifications for model optimization. For environments where the dynamic behavior of the gas–cluster–aerosol system differs from the standard assumptions, model predictions become distorted even if the chemical properties of the vapors and particles were perfectly accurately represented. The explicit model approach enables quantifying the role of the transient dynamic processes, thereby complementing steady-state laboratory experiments. We show that the dynamic couplings and their potential effects on aerosol number size distributions depend on available cluster-forming compounds. The presented model tool can be used for benchmark simulations of representative environments and chemistries for mapping of the direction and magnitude of these effects for given types of ambient conditions. This is necessary not only for seeking model–measurement closure in field studies, but also for assessing potential biases in computationally heavy large-scale models. Ultimately, such benchmark simulations provide possibilities to derive and test improved, optimal simplifications for representing secondary particle yields.

## Methods

### Molecular cluster and aerosol dynamics

Modeling the time evolution of cluster and aerosol distributions is based on the general dynamic equation (GDE). The time derivative of the concentration of aerosol particles of a given size is

1 $$\frac{\partial c\left(v,t\right)}{\partial t} = {\left.\frac{\partial c\left(v,t\right)}{\partial t}\right|}_{\mathrm{form}}+{\left.\frac{\partial c\left(v,t\right)}{\partial t}\right|}_{\mathrm{cond}-\mathrm{evap}}+{\left.\frac{\partial c\left(v,t\right)}{\partial t}\right|}_{\mathrm{coag} \, \mathrm{src}}-{\left.\frac{\partial c\left(v,t\right)}{\partial t}\right|}_{\mathrm{coag} \, \mathrm{sink}}-{\left.\frac{\partial c\left(v,t\right)}{\partial t}\right|}_{\mathrm{ext} \, \mathrm{sink}},$$

where *c*(*v*,*t*) is the number concentration density of particles of volume *v*, and the terms from left to right correspond to the formation of new particles from molecular clusters, growth or shrinkage due to vapor condensation and evaporation, formation from coagulation of smaller particles, loss due to coagulation with other particles, and loss due to external sinks. The terms in Eq. (1) can be written through the process rate constants as^40^

2 $$\frac{\partial c\left(v,t\right)}{\partial t}={J}_{0}\left(v\right)\delta \left(v-{v}_{0}\right)-\frac{\partial }{\partial v}\left(\frac{\mathrm{d}v}{\mathrm{d}t}\cdot c\left(v,t\right)\right)+\frac{1}{2}{\int }_{{v}_{0}}^{v-{v}_{0}}\beta \left(v-{v}^{{{\prime}}},{v}^{{{\prime}}}\right)c\left(v-{v}^{{{\prime}}},t\right)c\left({v}^{{{\prime}}},t\right)\mathrm{d}{v}^{{{\prime}}}-c\left(v,t\right){\int }_{{v}_{0}}^{\infty }\beta \left({v}^{{{\prime}}},v\right)c\left({v}^{{{\prime}}},t\right)\mathrm{d}{v}^{{{\prime}}}-S\left(v\right)c\left(v,t\right),$$

where *J*_0_ is the formation rate of new particles, d*v* / d*t* is the condensational growth rate, *β*(*v*,*v*’) is the coagulation rate constant between sizes *v* and *v*’, and *S*(*v*) is the external loss rate constant. The smallest particle size is *v*_0_, at which new particles are introduced. Equation (2) is the continuous form of the GDE, which is suitable for particles that have grown past the initial formation stage^15,40^.

The formation rate *J*_0_ follows from the molecular clustering processes that occur below size *v*_0_. These processes are described by the discrete cluster GDE, which is similar to Eq. (2) but treats the size distribution molecule-by-molecule instead of assuming a continuous concentration density *c*^40^:

3 $$\frac{\mathrm{d}{C}_{i}}{\mathrm{d}t}=\frac{1}{2}\sum_{j<i}\left({\beta }_{j,i-j}{{C}_{j}C}_{i-j}-{\gamma }_{i\to j,i-j}{C}_{i}\right)-\sum_{j}\left({\beta }_{i,j}{{C}_{i}C}_{j}{-\gamma }_{i+j\to i,j}{C}_{i+j}\right)-{S}_{i}{C}_{i.}$$

In Eq. (3), particle size is characterized by the exact molecular composition instead of volume, and d*C*_*i*_ / d*t* is the time derivative of the concentration of cluster *i* of a given composition. The first sum of terms includes all collisions between any clusters and / or molecules that produce cluster *i*, and the corresponding reverse evaporations in which the cluster is lost. The second sum covers losses of cluster *i* due to collisions with all other clusters and molecules, and formation of cluster *i* from the reverse evaporations of larger clusters. The last term is cluster removal due to external sinks. *β*_*i*,*i*_ is the collision rate constant between compositions *i* and *j*, *γ*_*i* → *j*,*i*−*j*_ is the evaporation rate constant of cluster *i* into compositions *j* and *i* − *j*, and *S*_*i*_ is the external loss rate constant.

*J*_0_ is given by the rate at which clusters grow beyond the cluster size regime:

4 $${J}_{0}=\frac{1}{2}\sum_{i}\sum_{j}{\beta }_{i,j}{C}_{i}{C}_{j} \quad \mathrm{ for }\,\,\left\{i+j|\mathrm{condition}\right\},$$

where the summation goes over all collisions that produce stable clusters outside of the cluster regime. The stability requirement, denoted by {*i* + *j* | condition}, means that the composition of the collision product *i* + *j* must satisfy given conditions. Some products can be expected to evaporate instantaneously, and therefore do not contribute to the formation rate but instead evaporate back to smaller clusters (Supplementary Information Sect. 1).

### Molecular cluster model plugin ClusterIn

In standard aerosol model set-ups, *J*_0_ is pre-determined for steady-state conditions (d*C*_*i*_ / d*t* = 0 in Eq. (3)), and there are no direct couplings between the cluster and aerosol regimes. Removing this discontinuity requires introducing the following couplings:

- The formation rate in Eq. (2) must follow directly from the time-dependent cluster concentrations *C*_*i*_ (Eqs. (3) and (4)).
- The cluster sink *S*_*i*_ in Eq. (3) includes cluster–aerosol coagulation and thus depends on the aerosol distribution. This scavenging process may also affect aerosol growth and thereby needs to be incorporated in Eq. (2).
- The condensation–evaporation term in Eq. (2) may transfer particles to the cluster regime, which needs to be considered by an additional cluster source term in Eq. (3).

To this end, we develop the cluster model plugin ClusterIn as an interface that simulates the cluster regime by solving the cluster GDE, and exchanges input and output with a host aerosol dynamics model which solves the aerosol GDE. The plugin enables the inclusion of the cluster size regime in any pre-existing aerosol modeling scheme without the need to build a separate discrete-sectional framework. The plugin is applied by calling it at each time step of the host model, as illustrated by Supplementary Fig. S1. The numerical simulation to solve the cluster concentrations is conducted with the cluster dynamics model ACDC^41,42^ which applies the VODE differential equation solver^43^. The details of the implementation of each cluster dynamics process (Table 1) are found in Supplementary Information Sect. 1.

Finally, it must be noted that while we refer to the size regimes simulated by the different models as cluster and aerosol regimes, there is no unambiguous threshold for such division. In general, the continuous aerosol GDE (Eq. 2) becomes a valid and computationally optimal approach as the initial clusters grow to sizes that are thermodynamically stable and consist of more than only a few molecules.

### Reference simulations with standard assumptions

The reference runs with no direct gas–cluster–aerosol couplings apply the steady-state particle formation rate (Supplementary Information Eq. S1), and new particles are set to have a constant size and composition (Supplementary Table S1). Concentrations of clustering vapors are reduced according to the number and assumed composition of newly-formed particles, but the reduction due to vapor-to-cluster sink is omitted. To avoid artificially high steady-state formation rates for strong acid–base mixtures, the concentration of H_2_SO_4_ is set to correspond to the sum of single H_2_SO_4_ molecules and single H_2_SO_4_ molecules with one or more base molecules attached to them^41^. This is in accordance with [H_2_SO_4_] measurements, which can include contributions from both free and base-containing H_2_SO_4_^44^. Cluster scavenging is considered by approximating the cluster sink based on the H_2_SO_4_ condensation sink CS as *S*_*i*_ = CS × (*d*_p,*i*_ / *d*_p,H2SO4_)^*m*^, where *d*_p,*i*_ and *d*_p,H2SO4_ are the diameters of cluster *i* and H_2_SO_4_, respectively^45^. The power-law function is derived for a lognormal aerosol size distribution and *m* is set to − 1.6, corresponding to a median aerosol diameter of 100 nm. This approximation does not require exact information on the aerosol distribution, but becomes less accurate for different distribution shapes. If aerosol particles shrink beyond the smallest size bin, a fraction of them is kept in the smallest bin and the remaining fraction is removed from the simulation.

### Cluster chemistries

The simulated cluster regimes include clusters consisting of up to (1) 6 H_2_SO_4_ and 6 NH_3_ molecules for cluster formation from H_2_SO_4_ and NH_3_, and (2) 4 H_2_SO_4_ and 4 DMA molecules for H_2_SO_4_ and DMA (Supplementary Table S1). Both chemistries cover electrically neutral and charged clusters, with ionization and recombination allowed to occur through collisions with generic charger ions^41^. The generic ion production rate is obtained as the sum of ionization due to galactic cosmic rays and radon decay^23^. Collision constants *β*_*i*,*i*_ are calculated as hard-sphere collision rates for electrically neutral collision parties, and according to the parameterization by Su and Chesnavich^46^ for collisions of neutral and charged species. Evaporation constants *γ*_*i* → *j*,*i*−*j*_ are obtained from quantum chemical formation free energies^41^. By default, we apply data computed at the DLPNO-CCSD(T)/aug-cc-pVTZ//ωB97X-D/6–31++G(d,p) level of theory by Besel et al.^36^ for H_2_SO_4_–NH_3_ and by Elm^47^ for H_2_SO_4_–DMA, and perform additional simulations using data computed at the RICC2/aug-cc-pV(T + d)Z//B3LYP/CBSB7 level^41^ (Supplementary Information Sect. 2).

### Trajectory model ADCHEM

The ADCHEM trajectory model setup is described in detail in the work by Roldin et al.^23^. In the present work, we run ADCHEM along 12 representative air mass trajectory cases (Supplementary Table S2; Supplementary Fig. S3), which were generated with the HYSPLIT trajectory model^48^. In addition to the benchmarked biogenic volatile organic compound gas-phase chemistry and secondary organic aerosol formation schemes^23^, the present ADCHEM version also includes a new dimethyl sulfide (DMS) and halogen multiphase chemistry mechanism^49^. This novel mechanism describes how DMS is oxidized in the gas and aqueous phases (in clouds and aerosol particles). For the marine air mass trajectory cases, the oceanic DMS emissions provide an important natural source of sulfuric acid and methanesulfonic acid (MSA). While H_2_SO_4_ contributes to both the formation and growth of new particles, MSA is treated as a condensable vapor that contributes to the modeled particle growth. DMA is treated as an effectively non-volatile condensable vapor, which is irreversibly lost from the gas phase to existing aerosol particles due to its strong binding to H_2_SO_4_^50^. Following the approach of previous studies^51,52^, DMA emissions are scaled according to NH_3_ emissions, assuming a scaling factor of 0.01. Particle number size distribution is represented by 100 logarithmically spaced particle size bins (sections) covering the diameter range from ca. 1 nm to 10 μm. Aerosol growth by condensation and coagulation is represented by a particle mass and number concentration conserving quasi-stationary size distribution scheme^22^.

For simplicity and computational efficiency, the model represents the lowermost 2500 m of the atmospheric column by two vertical model layers (boxes), analogous to the model setup described by Tunved et al.^53^. The first atmospheric layer represents a well-mixed boundary layer that extends from the ground to the altitude defined by the planetary boundary layer height (PBLH). The PBLH along the trajectories is obtained from the HYSPLIT model runs. The second atmospheric layer extends from the PBLH to 2500 m above the ground, and represents the residual layer. The composition in the first layer changes when the PBLH increases during the day as air from the residual layer is entrained into the mixed layer. When the PBLH decreases during night, a fraction of air in the mixed layer merges into the residual layer.

### Adiabatic cloud parcel model

An adiabatic cloud parcel model^27^ was used to calculate the number of cloud condensation nuclei, i.e. activated cloud droplets, as described by Roldin et al.^23^. The cloud parcel model was applied at the end of each trajectory using the aerosol particle number size distribution and chemical composition from the mixed layer as input. CCN concentrations were calculated for different updraft velocities ranging from values corresponding to stratiform clouds (≲0.5 m s^−1^) to cumulus clouds (≳1 m s^−1^).

## Supplementary Information

Supplementary Information.

## Data Availability

Data used in simulations are available from the corresponding author upon reasonable request.

## Code availability

The ClusterIn plugin is available at https://github.com/tolenius/ClusterIn. All codes used for simulations and analysis are available from the corresponding author upon reasonable request.
