# Supplementary material for: Role of gas–molecular cluster–aerosol dynamics in atmospheric new-particle formation
Source: Sci Rep. 2022 Jun 16;12:10135. doi: 10.1038/s41598-022-14525-y (PMC9203563; doi:10.1038/s41598-022-14525-y)
Supplement: Supplementary file 1 — Supplementary Information. [file 41598_2022_14525_MOESM1_ESM.pdf]

Supplementary Information for:

Role of gas–molecular cluster–aerosol dynamics in atmospheric new-particle formation

Tinja Olenius and Pontus Roldin

1 Supplementary information on ClusterIn

The coupling of the ClusterIn plugin to an aerosol dynamics model and the workflow between the two models is illustrated in Figure S1. The cluster regime modeled by ClusterIn consists of a set of discrete cluster compositions, as demonstrated by Figure S2. The exact cluster set depends on the chemical system and available thermochemical data, which is in practice obtained by quantum chemistry. Compositions that are highly unstable, such as acid–base clusters with a very low acid:base ratio, can be excluded[1]. The sets applied in this work are listed in Supplementary Information Section 2. The molecular-resolution cluster GDE for the given cluster set is generated and solved by the ACDC model[1,2], which is embedded in ClusterIn. The treatment of each cluster dynamics process or cluster–aerosol coupling is detailed in Supplementary Information Sections 1.1–1.5 below. Technical details of setting up the plugin for a selected set of clusters and quantum chemical data are given together with the ClusterIn program.

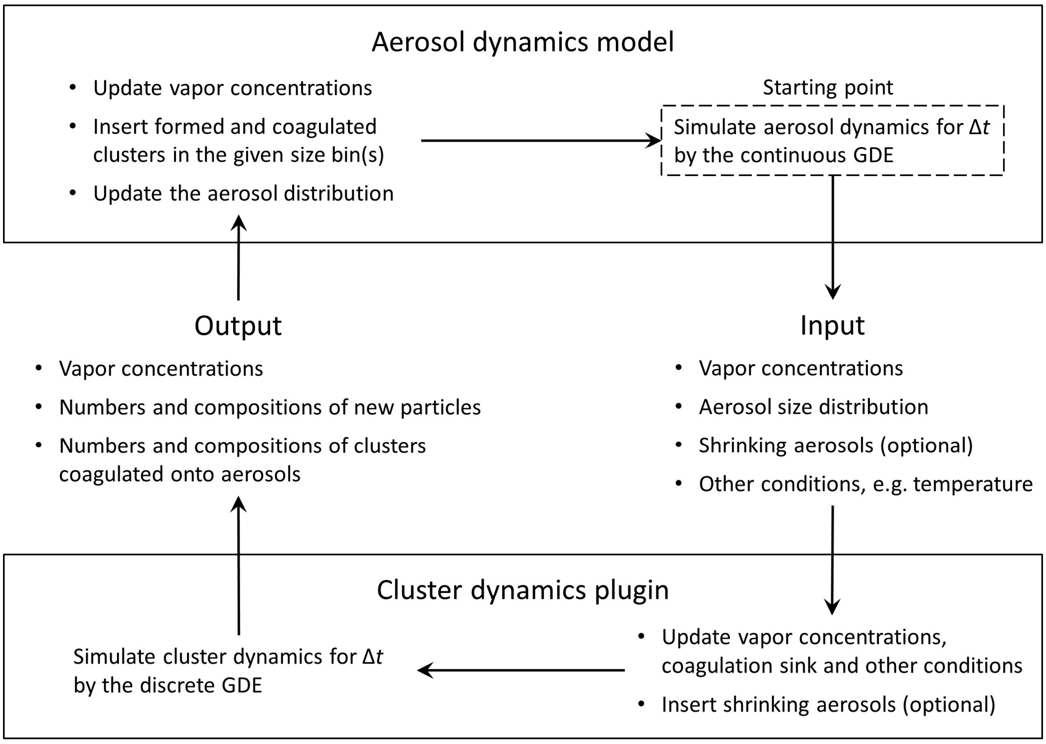

Figure S1: A flowchart depicting the input–output flow between the host aerosol model and the cluster dynamics plugin ClusterIn during a model time step Δt. The dashed box marks the starting point of the cycle.

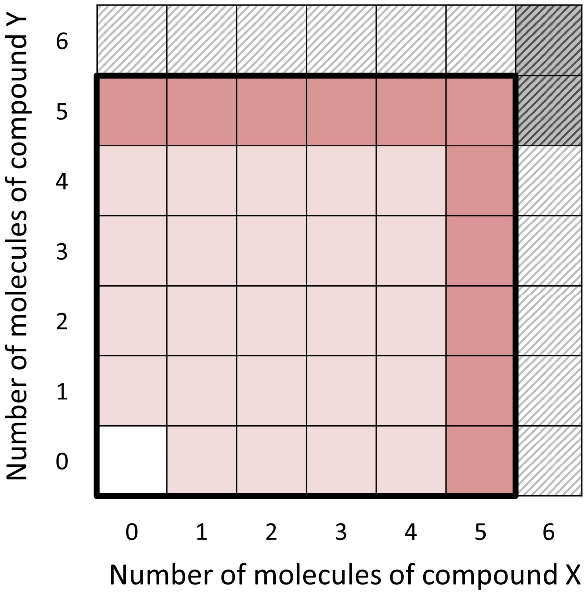

Figure S2: Example of a set of cluster compositions included in the cluster regime for a 2-component chemical system. The red area inside the black rectangle corresponds to modeled clusters, and the grey raster area outside of the rectangle to clusters beyond the modeled cluster regime. Stable compositions that are allowed to grow into the aerosol regime are colored with dark grey, and unstable compositions that evaporate back to the cluster regime are colored with light grey. The dark red area corresponds to compositions located at the boundaries of the cluster regime. Shrinking aerosol particles are placed in the boundary composition that most closely corresponds to the aerosol molar composition.

1.1 Time evolution of cluster concentrations

The time evolution of the cluster concentrations is explicitly simulated for each time step of the aerosol model. After the step, the concentrations are saved and used as initial values at the next call. The main input parameters include the vapor concentrations, the temperature, information on the aerosol size distribution, and possibly also the ambient ion production rate in case that the simulated clusters include charged species.

The number and composition of clusters that grow out of the cluster size regime during the time step are returned to the aerosol model. Here, the cluster concentrations as well as the formation rate evolve from the ambient conditions of the previous time step to those of the current step, and are not necessarily in a steady state at any point. Mathematically, the number of newly-formed particles defined by the steady-state approach is written as

$$\Delta C_{\mathrm{new,ss}} = J_{\mathrm{ss}}\Delta t = \left(\frac{1}{2}\sum_i \sum_j \beta_{i,j} C_{\mathrm{ss},i} C_{\mathrm{ss},j}\right)\Delta t \text{ for } \{i+j|\text{condition}\}, \quad \text{(S1)}$$

where J_{ss} is the steady-state formation rate and Δt is the time step, and the summation goes over all possible cluster–molecule and cluster–cluster collisions that lead to growth out of the cluster regime satisfying the stability conditions. $C_{\mathrm{ss},i}$ and $C_{\mathrm{ss},j}$ are the steady-state concentrations of the colliding parties i and j. By contrast, the explicitly simulated number of new particles is

$$\Delta C_{\mathrm{new}} = \int_t^{t+\Delta t} \left(\frac{1}{2}\sum_i \sum_j \beta_{i,j} C_i C_j\right) \mathrm{d}t \text{ for } \{i+j|\text{condition}\}, \quad \text{(S2)}$$

where the integrand is otherwise as in Eq. (S1), but C_i and C_j are now time-dependent concentrations.

1.2 Effect of cluster formation on vapor concentrations

The concentrations of vapors participating in cluster formation are received as input, and their time evolution during the clustering is simulated together with that of the molecular clusters. Vapor concentrations at the end of the simulated time step are returned to the aerosol model, thus accounting for changes due to vapor–cluster exchange and binding of vapor molecules in clusters. While standard aerosol model set-ups allow reducing the vapors by the amount of molecules bound in the newly-formed particles, such reduction does not include the molecules bound in clusters below the lower limit of the aerosol size range.

1.3 Sizes and compositions of newly-formed particles

As clusters can grow out of the cluster regime through different cluster–molecule or cluster–cluster collisions, the collision products which are transferred to the aerosol regime may have slightly different sizes and compositions. This is relevant for sectional, logarithmically spaced aerosol models of high size resolution, as the first size bins can be very narrow. ClusterIn tracks the numbers and compositions of the product clusters, distributes them in the aerosol size bins according to the bin limits, and returns for each bin n the number of new particles $\Delta C_{\mathrm{new},n}$ (Eq. (S2)) and their composition

$$N_{\mathrm{new},n,k} = \frac{\sum_i (\Delta C_{\mathrm{new},n,i} N_{i,k})}{\Delta C_{\mathrm{new},n}}, \tag{S3}$$

where $N_{\mathrm{new},n,k}$ is the average number of molecules k in clusters grown to bin n, and the summation goes over all clusters i that are placed in bin n. $\Delta C_{\mathrm{new},n,i}$ is the number of clusters of composition i, and $N_{i,k}$ is the number of molecules k in cluster i. As most aerosol models do not separate between possible different charging states, electrically neutral and charged clusters are by default lumped in ClusterIn output. Charged cluster fractions can be returned when needed.

1.4 Cluster scavenging by aerosol particles

Scavenging of clusters by larger aerosols is a central factor governing particle formation dynamics. While some formation rate data include the effect of approximative scavenging sinks on the formation rate[3], the scavenged clusters are lost from the cluster–aerosol system. ClusterIn determines the scavenging rate constant of each cluster onto each aerosol size bin, and returns the number of clusters scavenged during the simulation time step. Vapors are not scavenged within ClusterIn, as vapor condensation onto aerosol particles is modeled within the host model.

The diameters and concentrations of the aerosol bins are passed to ClusterIn, which calculates the cluster–aerosol coagulation rate constants according to Fuchs-Sutugin formulae[4], or alternatively by a user-defined routine. The total sink for each cluster (S_i in Eq. (3)), applied in the cluster simulation, is obtained by summing the cluster scavenging rates over all aerosol bins. After the time step, the scavenged cluster concentrations are mapped back onto the bins according to the bin-specific scavenging constants. Mathematically, the number of clusters of composition i scavenged by bin n is thus written as

$$\Delta C_{n,i} = \frac{\beta_{n,i} C_n}{\sum_n (\beta_{n,i} C_n)} \Delta C_{\mathrm{scav},i} = \frac{\beta_{n,i} C_n}{\sum_n (\beta_{n,i} C_n)} \int_t^{t+\Delta t} S_i C_i \,\mathrm{d}t, \tag{S4}$$

where $\beta_{n,i}$ is the coagulation constant between cluster i and aerosol bin n, and C_n is the concentration in bin n. $\Delta C_{\text{scav},i}$ is the number of clusters i that are scavenged during the time step according to the rate constant

$$S_i = \sum_n(\beta_{n,i}C_n). \tag{S5}$$

Within the aerosol model, the scavenged clusters can be distributed to the aerosol bins similarly to aerosol–aerosol coagulation by utilizing the coagulation routine of the host model. Another option is to further simplify the ClusterIn output by returning the total numbers of scavenged molecules for each compound and each bin instead of the numbers of clusters. The number of molecules of compound k transferred to bin n is

$$\Delta C_{n,k} = \sum_i\left(\Delta C_{n,i}N_{i,k}\right), \tag{S6}$$

where the summation goes over all scavenged clusters i. The shift in the aerosol size distribution due to addition of molecules $\Delta C_{n,k}$ can now be taken into account by using the condensation routine of the aerosol model. Here, it is assumed that coagulation of very small clusters onto larger particles can be treated similarly to vapor condensation. In the present simulations, we incorporate scavenged clusters similarly to coagulation, and test the condensation-like treatment in additional simulations (Supplementary Figure S11).

In addition to the coagulation sink, the host model can also give other types of sinks to the plugin through a user-defined subroutine. This enables including for clusters the same external sinks that are applied for aerosols, such as deposition onto surfaces. Here, we apply the dry and wet deposition as implemented in the ADCHEM model[5].

1.5 Aerosol evaporation beyond the smallest size covered by the aerosol model

ClusterIn allows the transfer of evaporating aerosol particles back to the cluster regime through passing the number and molecular composition of the shrinking particles to the plugin. The exact cluster composition within ClusterIn is determined as follows: the molecular composition is converted to a molar composition, which is compared to the molar compositions of the largest clusters included in the cluster regime. The largest clusters are those for which the number of molecules equals the maximum number of molecules possible in any cluster at least for one component, that is, the clusters at the system boundary, as depicted in Supplementary Figure S2. The closest cluster composition is determined as that corresponding to the minimum difference to the input molar composition, defined as

$$\Delta n_{\text{sum,min}} = \min_i \sum_k\left|n_{i,k} - n_{\text{input},k}\right|, \tag{S7}$$

where the differences in the mole fractions $n_{i,k}$ and $n_{\text{input},k}$ of component k in the boundary cluster i and in the input composition, respectively, are summed over all components. The number concentration of the evaporating particles is added to that of the target cluster, and possible differences in the absolute molecular composition are accounted for by adjusting the vapor concentrations for each component k by

$$\Delta C_{\text{vapor},k} = C_{\text{input}}\left(N_{\text{cluster},k} - N_{\text{input},k}\right), \tag{S8}$$

where C_{input} is the concentration of the evaporating particles, and $N_{cluster,k}$ and $N_{input,k}$ are the numbers of molecules of component k in the target cluster and the input particles, respectively.

The reason for using the molar composition is to avoid conflicting overlaps in the description of particle dynamics and thermodynamics by the cluster and aerosol models. If the aerosol model, for instance, gives a molecular composition corresponding to a very small cluster well beyond the boundary of the cluster regime, the prediction cannot be considered reliable as such clusters are not correctly described by the continuous GDE and the macroscopic thermodynamics applied by the aerosol model. Therefore, the evaporating particles are placed at the boundary of the cluster and aerosol regimes, and their further time evolution is modeled by ClusterIn.

2 Quantum chemistry data sets

The main simulations apply the quantum chemistry data sets by Besel et al.[6] and Elm[7] for H_2SO_4–NH_3 and H_2SO_4–DMA clusters, respectively, computed at the DLPNO-CCSD(T)/aug-cc-pVTZ//ωB97X-D/6-31++G(d,p) level of theory. This is considered the current best method for atmospheric molecular clusters. To confirm that the qualitative results are independent of the quantitative cluster data, test simulations are conducted applying a previous data set computed with the RICC2/aug-cc-pV(T+d)Z//B3LYP/CBSB7 method[1]. Tests with the H_2SO_4–NH_3–DMA cluster set apply RICC2 data[8], which is thus far the only existing comprehensive data set for the 3-component chemical system. Cluster compositions included in the sets and the stability criteria for allowing clusters to grow into the aerosol model regime are listed in Table S1.

While the different quantum chemistry methods give qualitatively similar results for, for example, differences in clustering efficiency between H_2SO_4–NH_3 and H_2SO_4–DMA chemistries, they differ in the quantitative cluster stabilities. In general, RICC2 tends to underpredict cluster evaporation, and DLPNO tends to overpredict it[9]. Both methods compare reasonably well with laboratory measurements in terms of trends and magnitudes of steady-state formation rates[6,10,8,11]. Therefore, the two methods can be roughly considered as upper- and lower-limit estimates for cluster formation.

Table S1: Cluster sets applied in this work. The sets cover clusters up to the stated molecular content, with highly unstable compositions excluded. Criteria for clusters to grow beyond the cluster model regime are given as minimum numbers of molecules that the grown cluster must contain. The criteria are different for electrically neutral, negative and positive clusters due to differences in their stabilities. For charged clusters, the molecule numbers include charged forms, that is, the H_2SO_4 number includes deprotonated acid HSO_4^-, and the base number includes protonated base with H+. Compositions written in bold correspond to the assumed composition of new particles in the standard simulation runs with no explicit cluster–aerosol dynamics.

**Data set**		**Maximum numbers of molecules in modeled clusters**	**Minimum numbers of molecules in clusters that grow to aerosol model regime**
DLPNO	H_2SO_4–NH_3	6 H_2SO_4, 6 NH_3	**7 H_2SO_4 + 6 NH_3** (neutral)<br>7 H_2SO_4 + 3 NH_3 (negative)<br>6 H_2SO_4 + 7 NH_3 (positive)
	H_2SO_4–DMA	4 H_2SO_4, 4 DMA	**5 H_2SO_4 + 4 DMA** (neutral)<br>5 H_2SO_4 + 2 DMA (negative)<br>3 H_2SO_4 + 5 DMA (positive)
RICC2	H_2SO_4–NH_3	5 H_2SO_4, 5 NH_3	**6 H_2SO_4 + 5 NH_3** (neutral)<br>6 H_2SO_4 + 3 NH_3 (negative)<br>5 H_2SO_4 + 6 NH_3 (positive)
	H_2SO_4–DMA	4 H_2SO_4, 4 DMA	**5 H_2SO_4 + 4 DMA** or 4 H_2SO_4 + 5 DMA (neutral)<br>5 H_2SO_4 + 2 DMA (negative)<br>3 H_2SO_4 + 5 DMA (positive)
	H_2SO_4–NH_3–DMA	4 H_2SO_4, 4 base (NH_3 or DMA)	5 H_2SO_4 + 4 base or 4 H_2SO_4 + 5 DMA; **5 H_2SO_4 + 3 NH_3 + 1 DMA** (neutral)<br>5 H_2SO_4 + 2 base (negative)<br>4 H_2SO_4 + 5 base or 3 H_2SO_4 + 5 DMA (positive)

3 HYSPLIT trajectories for ADCHEM simulations

The ADCHEM model was run along 4-day trajectories from the Hybrid Single Particle Lagrangian Integrated Trajectory Model (HYSPLIT)[12]. The trajectories are shown in Figure S3, and the ambient conditions along the trajectories are summarized in Table S2 and Figure S4 (for more information, see the work by Roldin et al.[3]). The selection of trajectories was chosen to include various types of conditions from marine to continental environments with varying levels of anthropogenic influence.

The gas and particle concentrations are initialized as follows: first, a preliminary simulation is conducted with low initial concentrations corresponding to clean ambient conditions. Second, the final simulation is run by setting the initial concentrations to correspond to half of those obtained at the end of the preliminary run. The purpose of this treatment is to apply concentrations and particle-phase composition

that are realistic for air masses that have been traveling over the modeled areas. The preliminary simulation results are also used as a test case to ensure that the overall results on gas–cluster–aerosol dynamics effects are not sensitive to the initial conditions.

Table S2: Summary of the main environmental conditions along the simulated trajectories.

Trajectory no.	Arrival time at the SMEAR II station (YYYYMMDDHH)	Environment types and locations
1	2014042421	Marine, remote continental (Arctic Ocean, Fennoscandia, Baltic Sea)
2	2014042200	Marine, remote continental (North Atlantic, Fennoscandia, Baltic Sea)
3	2014041509	Marine, continental, anthropogenic influence (British Isles, North Sea, Baltic Sea)
4	2013051703	Marine, continental, anthropogenic influence (British Isles, North Sea, Baltic Sea)
5	2014041915	Continental, marine, anthropogenic influence (Northern Germany, Fennoscandia, Baltic Sea)
6	2014041900	Continental, marine, anthropogenic influence (Fennoscandia, Northern Poland, Baltic Sea)
7	2014042106	Continental, marine, anthropogenic influence (Fennoscandia, Baltic Sea)
8	2013052512	Continental, anthropogenic influence (Belarus, Western Russia)
9	2013052509	Continental, anthropogenic influence, remote (Western Russia)
10	2013051912	Continental, remote (Western Russia)
11	2013052006	Continental, remote, anthropogenic influence (Kola Peninsula, Western Russia)
12	2014050115	Continental, remote, anthropogenic influence (Kola Peninsula, Northern Finland)

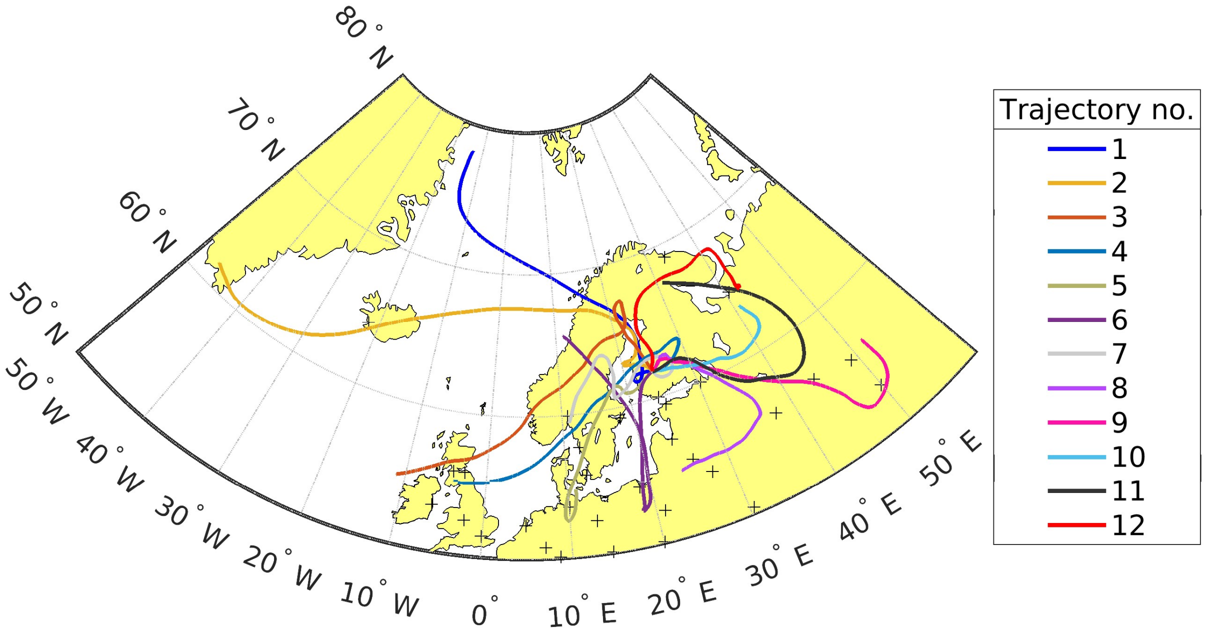

Figure S3: HYSPLIT air mass trajectories used for the ADCHEM model simulations. Major cities are marked with black crosses.

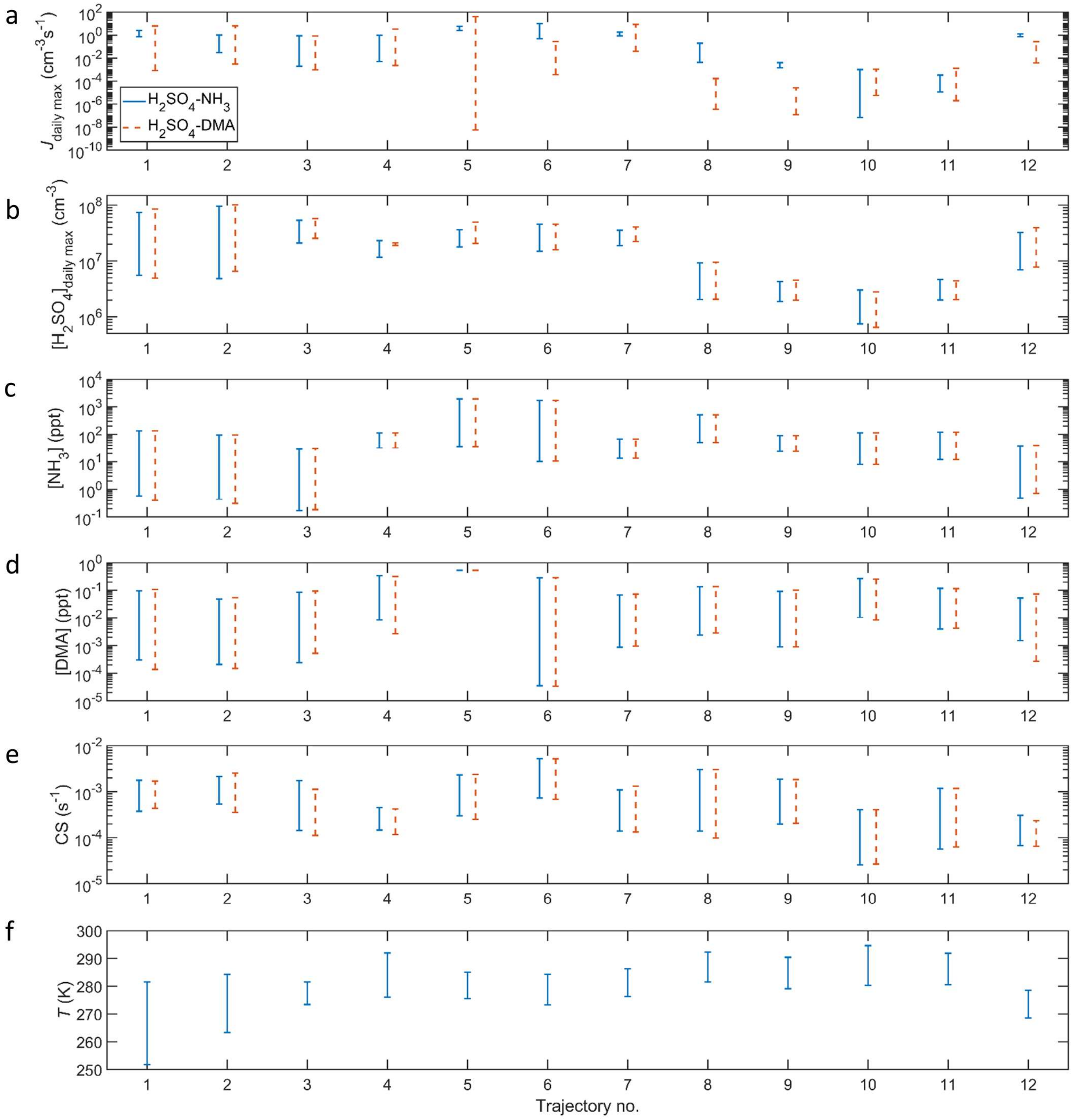

Figure S4: Ranges of ambient conditions that characterize new-particle formation along the simulated trajectories. Vertical bars give the minimum and maximum values in the explicit simulation for each trajectory and assumed cluster formation chemistry. For parameters that typically exhibit strong diurnal pattern and very low values in nighttime (J and [H_2SO_4]), ranges of daily maximum values are given. Panel (a): formation rate J; panels (b)-(d): concentrations of clustering vapors; panel (e): condensation sink (here calculated for H_2SO_4; characterizes also the order of magnitude of cluster scavenging sink); panel (f): temperature.

4 Additional ADCHEM simulations

4.1 Test simulations with different input settings

Results of test simulations assessing the sensitivity of the $C_{standard}$ vs. $C_{explicit}$ comparison to model input and ambient conditions are summarized below. The comparisons are shown as scatter plots of particle numbers similarly to panels (a) and (c) in Figure 4 in the main text. To avoid redundant figures, not all test cases are shown if they are very similar to the other figures.

Increasing DMA emissions by a factor of 10 with no other changes in the setup leads to less but yet significant overprediction of particle numbers (by factors of up to ca. 10; Figure S5). Here, maximum DMA concentrations are increased by ca. an order of magnitude compared to the default case. While amine emissions are generally not well quantified, both concentration levels are of the order of previous model and measurement assessments for the simulated locations[13,14].

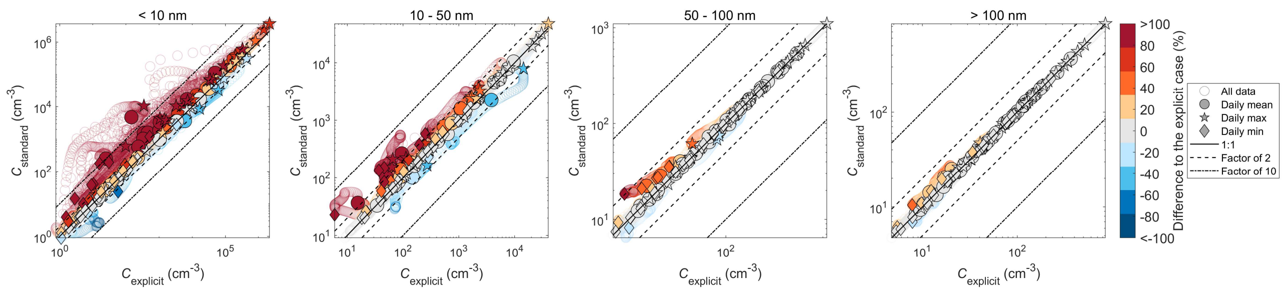

Figure S5: Size-classified particle number concentrations for the standard and explicit cases for all trajectories with initial particle formation from H_2SO_4–DMA, with DMA emissions increased by a factor of 10 compared to the default case shown in Figure 4 in the main text.

Simulations with very low initial gas and particle concentrations (Supplementary Information Section 3) give results similar to the default case (not shown). H_2SO_4–DMA cluster formation shows somewhat larger effects compared to Figure 4 in the main text, also at the largest particle sizes, as the influence of new-particle formation is enhanced at low particle numbers.

Results for simulations applying the RICC2 quantum chemistry data set (Supplementary Information Section 2) are shown in panels (a) and (b) of Figure S6. The $C_{standard}$ vs. $C_{explicit}$ comparison is similar to the DLPNO-based simulations, with even larger effects for H_2SO_4–DMA as the RICC2 method generally predicts stronger clustering than the DLPNO method (Supplementary Information Section 2). The RICC2-based results for the 3-component H_2SO_4–NH_3–DMA system are presented in panel (c). The overprediction is less pronounced than for H_2SO_4–DMA cluster formation due to also NH_3 acting as a clustering agent. However, the overprediction tendency persists, with $C_{standard}$ being higher than $C_{explicit}$ by factors of up to ca. 10 at the smallest sizes. It should be noted that the 3-component cluster system is a less studied and less benchmarked clustering chemistry than the 2-component systems, and the results should thus be considered tentative.

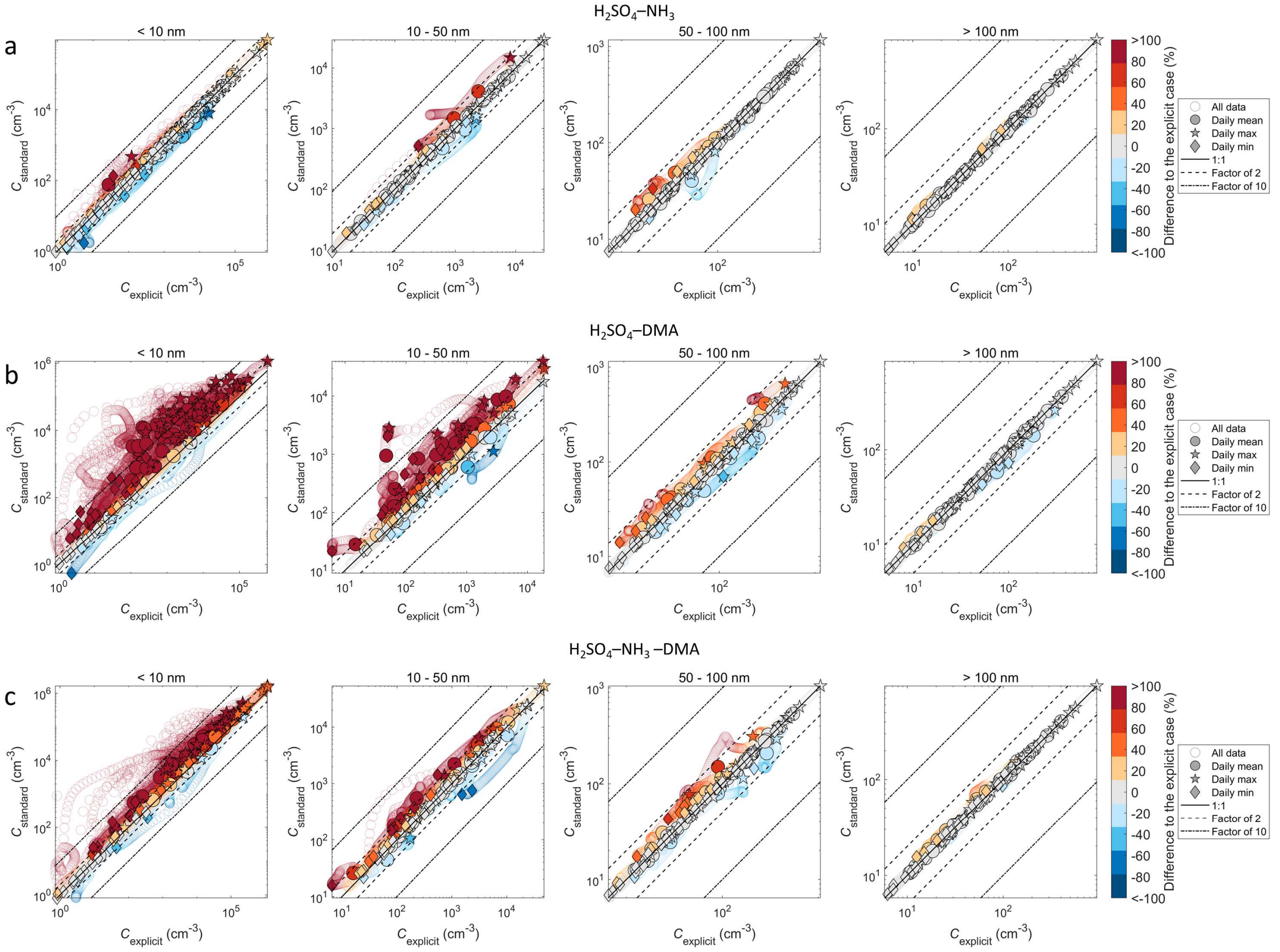

Figure S6: Size-classified particle number concentrations for the standard and explicit cases for all trajectories with initial particle formation from H_2SO_4–NH_3 (panel (a)), H_2SO_4–DMA (panel (b)), and H_2SO_4–NH_3–DMA (panel (c)) for simulations using the RICC2 quantum chemistry data set for cluster evaporation rates.

Finally, representative polluted conditions were tested by increasing all anthropogenic emissions (CO, NO_2, SO_2, NH_3, VOC, PM) by a factor of 10. To ensure that also [DMA] becomes significantly higher despite the increased condensation sink caused by the increased emissions, an additional scaling factor of 20 was added for all DMA emissions in simulations with H_2SO_4–DMA cluster formation. For H_2SO_4–NH_3 clustering, the results are similar to the default conditions: the differences between $C_{standard}$ and $C_{explicit}$ are mainly within a factor of 2, with somewhat larger effects at sizes of < 10 nm (Figure S7). For H_2SO_4–DMA, the magnitude of overprediction is lower compared to the default case. However, the trajectories still cover also periods with lower [DMA], and in addition low [H_2SO_4] and H_2SO_4 reduction may cause similar overprediction effects as DMA reduction (Figure S9 and Figure S10). This is demonstrated in Figure S8, which shows the time series for trajectory 10 with increased emissions, including periods of $[H_2SO_4]_{standard} > [H_2SO_4]_{explicit}$ and $J_{standard} > J_{explicit}$.

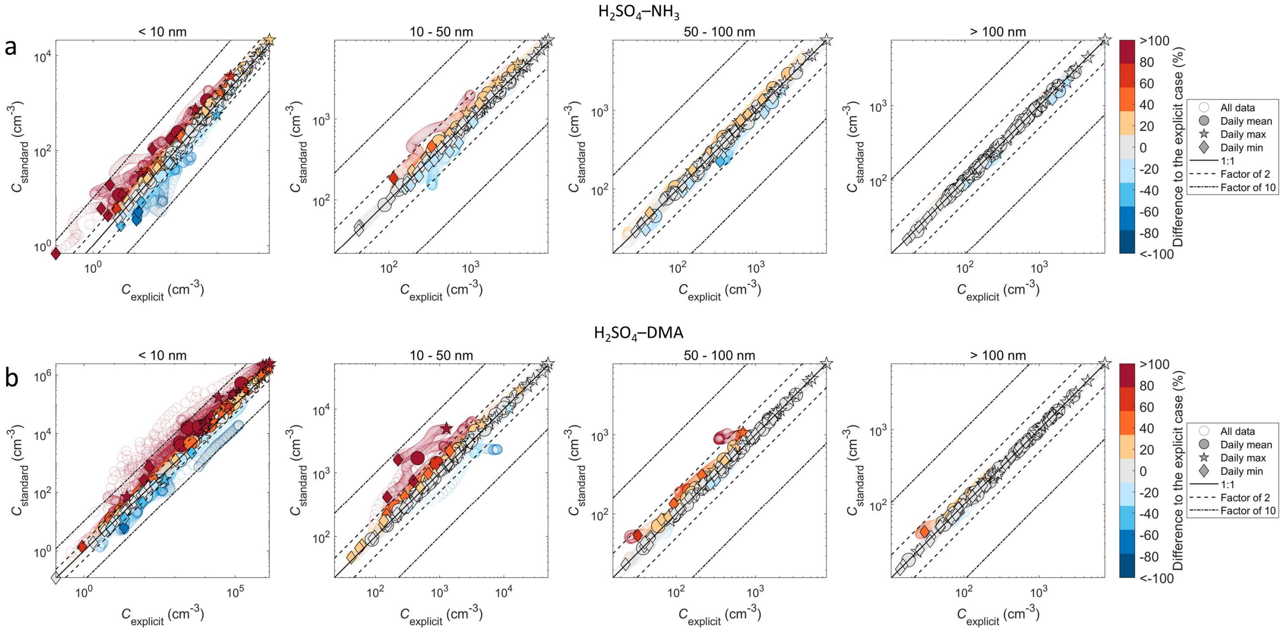

Figure S7: Size-classified particle number concentrations for the standard and explicit cases for all trajectories with initial particle formation from H_2SO_4–NH_3 (panel (a)), and H_2SO_4–DMA (panel (b)), with increased anthropogenic emissions.

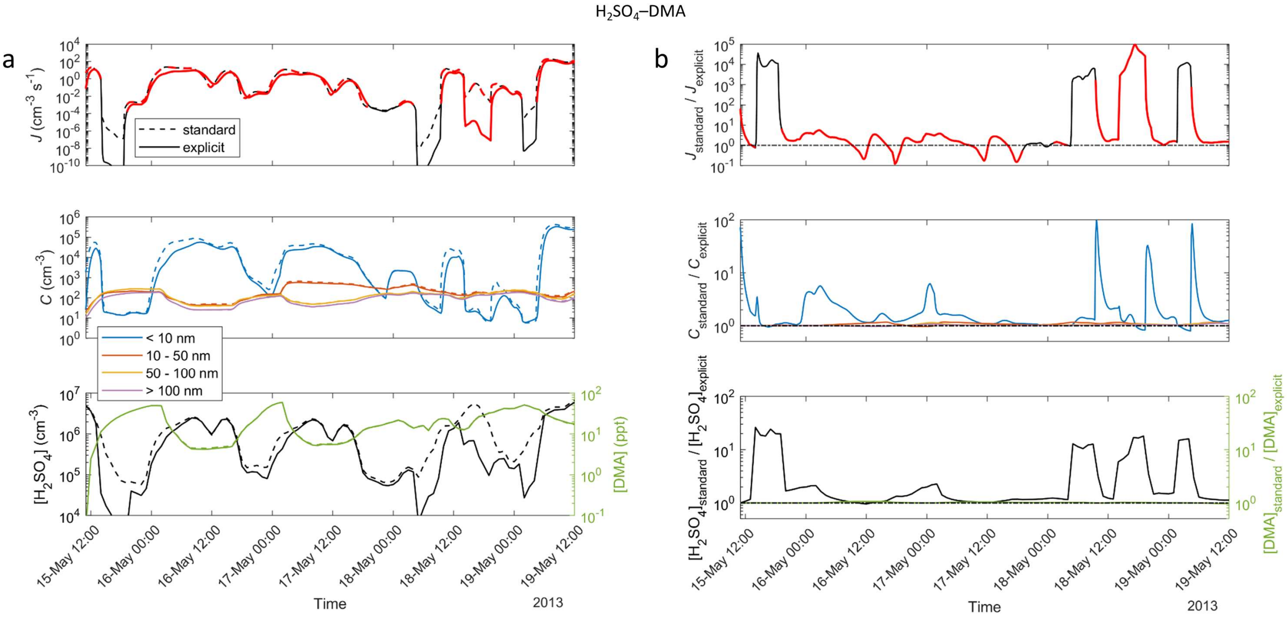

Figure S8: Panel (a): Particle formation rate (J), size-classified particle concentrations (C) and concentrations of clustering vapors along trajectory 10 (UTC time) for the standard and explicit simulation cases with initial particle formation from H_2SO_4–DMA and increased anthropogenic emissions. Panel (b): Ratio between the standard and explicit cases. Periods during which $J \geq 10^{-3}$ cm^{-3} s^{-1} for at least one of $J_{standard}$ and $J_{explicit}$ are marked with red in the time series of J.

4.2 Assessment of the effects of different dynamic processes

Table S3 clarifies the details of the ADCHEM test simulations in which the roles of different cluster dynamics processes are studied (Section 2.3 in the main text). The test set-ups are constructed according to the following reasoning: we start from the standard case, and first include the time-dependent cluster and vapor dynamics (test 1). In this way, nothing else is changed, allowing the assessment of non-steady-state effects compared to the standard approach. The significance of interactions between the cluster and aerosol distributions (tests 2–4), on the other hand, is most straight-forwardly assessed by removing one interaction at a time from the explicit case.

Table S3: Details of the set-ups used for test simulations for studying the effects of each cluster dynamics process. The effects are assessed by comparing the reduced simulation case, from which the dynamic feature is removed, to the reference case which includes the feature.

Test no.	Purpose	Dynamic process	Set-up	Compared cases	
				**Reduced case**	**Reference case**
1	Isolating the effect of the steady-state assumption	Time evolution of cluster concentrations and effect of cluster formation on vapor concentrations	As for the standard case, but without the steady-state assumption with vapor concentrations simulated together with cluster concentrations within ClusterIn	Standard case	Test 1
2	Assessing the importance of each cluster–aerosol interaction	Sizes and compositions of newly-formed particles	As for the explicit case, but assuming constant size and composition of new particles	Test 2	Explicit case
3		Cluster scavenging by aerosol particles	As for the explicit case, but excluding transfer of scavenged clusters to aerosol bins	Test 3	Explicit case
4		Aerosol evaporation beyond the smallest size covered by the aerosol model	As for the explicit case, but excluding aerosol evaporation to cluster regime	Test 4	Explicit case

The overprediction of H_2SO_4–DMA particle formation is likely to be linked to high-biased DMA concentrations in the standard case in which vapor concentrations are not affected by clustering (while [H_2SO_4] is less altered; Figure S9). To verify that vapor–cluster dynamics play an important role for the H_2SO_4–DMA case, we performed test simulations omitting these dynamics. Here, the set-up was otherwise similar to the explicit case and no steady state was assumed for cluster concentrations, but vapor concentrations were set to constant values during clustering. The results, presented in Figure S10, show that while also the time evolution of the cluster concentrations and the cluster–aerosol interactions affect the formation process, vapor–cluster couplings and reduction of vapor are a major factor behind the overprediction.

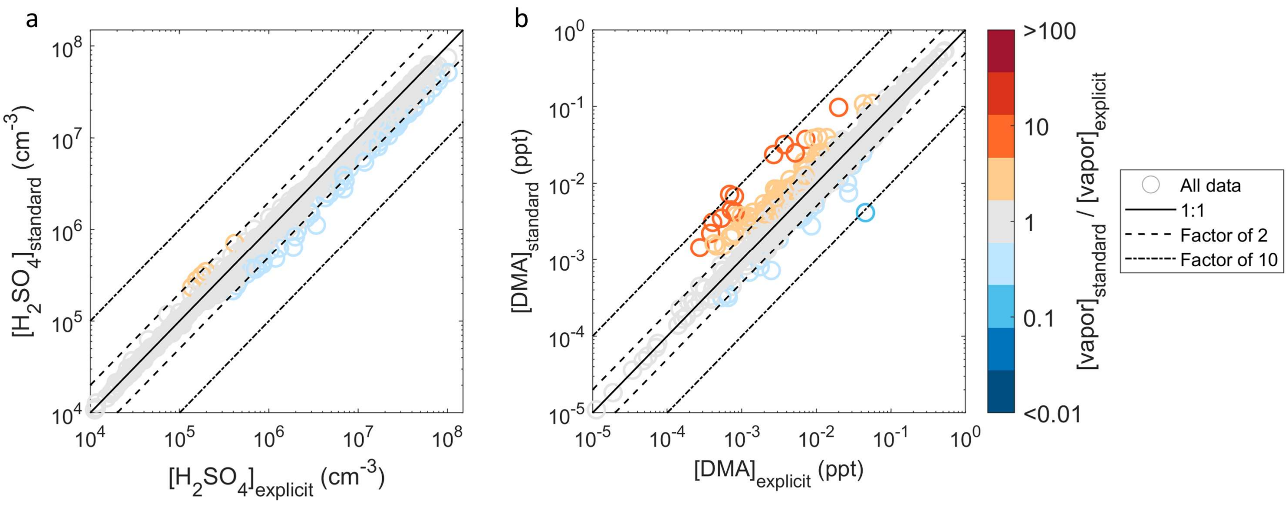

Figure S9: Concentrations of H_2SO_4 (panel (a)) and DMA(panel (b)) for the standard and explicit cases for all trajectories with initial particle formation from H_2SO_4–DMA. The color scale gives the ratio between the standard and explicit cases.

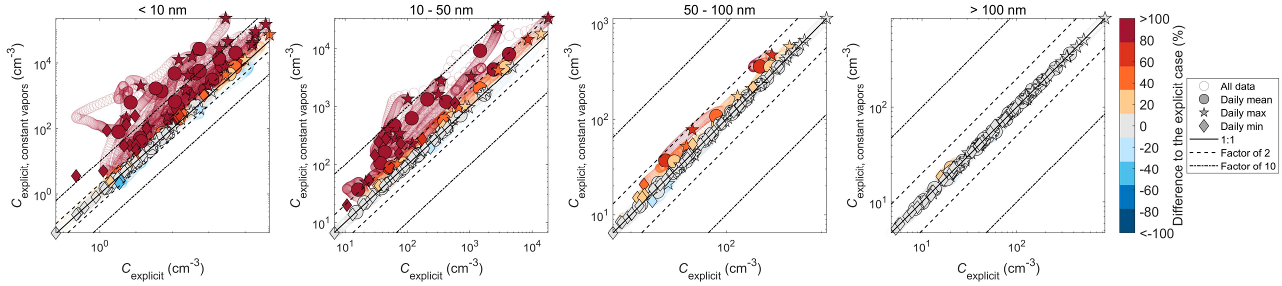

Figure S10: Size-classified particle number concentrations for all trajectories with initial particle formation from H_2SO_4–DMA for the following cases: (1) the explicit case, and (2) the explicit case with vapor concentrations set to constant values during cluster formation, i.e. with vapor–cluster interactions and vapor-to-cluster sink omitted.

We also tested treating the effect of cluster–aerosol coagulation on aerosol growth similarly to condensation (Supplementary Information Section 1.4), as shown in Figure S11. The results are close to those obtained by the explicit coagulation treatment, suggesting that the approximation is reasonable. The condensation-like treatment reduces code complexity as it only requires adding the given amount of each vapor species in the aerosol size bins, instead of looping through all collisions between each cluster composition and aerosol bin.

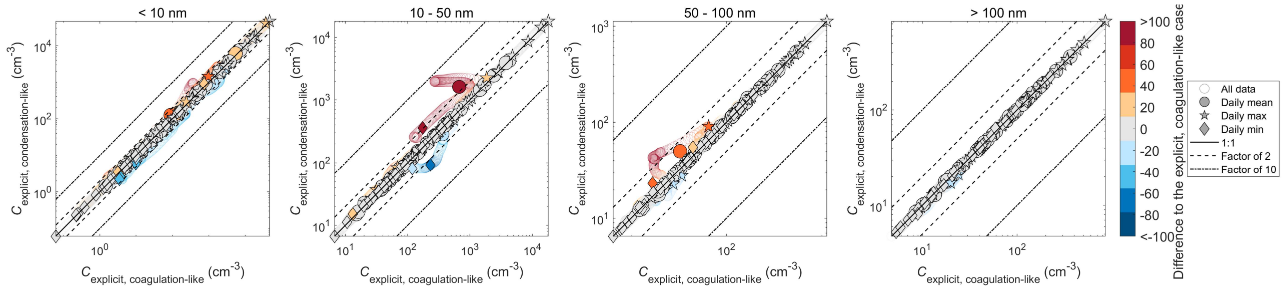

Figure S11: Size-classified particle number concentrations for all trajectories with initial particle formation from H_2SO_4–DMA for the following cases: (1) the explicit case, and (2) the explicit case with aerosol growth due to cluster–aerosol coagulation treated similarly to condensation.

References

1. Olenius, T., Kupiainen-Määttä, O., Ortega, I. K., Kurtén, T. & Vehkamäki, H. Free energy barrier in the growth of sulfuric acid–ammonia and sulfuric acid–dimethylamine clusters. *J. Chem. Phys.* **139**, 084312 (2013).

2. Olenius, T. *Atmospheric Cluster Dynamics Code: Software repository*. https://github.com/tolenius/ACDC (2021).

3. Roldin, P. *et al.* The role of highly oxygenated organic molecules in the Boreal aerosol-cloud-climate system. *Nat. Commun.* **10**, 4370 (2019).

4. Seinfeld, J. H. & Pandis, S. N. *Atmospheric chemistry and physics: From air pollution to climate change*. (John Wiley & Sons, Inc., 2006).

5. Roldin, P. *et al.* Development and evaluation of the aerosol dynamics and gas phase chemistry model ADCHEM. *Atmospheric Chem. Phys.* **11**, 5867–5896 (2011).

6. Besel, V., Kubečka, J., Kurtén, T. & Vehkamäki, H. Impact of Quantum Chemistry Parameter Choices and Cluster Distribution Model Settings on Modeled Atmospheric Particle Formation Rates. *J. Phys. Chem. A* **124**, 5931–5943 (2020).

7. Elm, J. An Atmospheric Cluster Database Consisting of Sulfuric Acid, Bases, Organics, and Water. *ACS Omega* **4**, 10965–10974 (2019).

8. Almeida, J. *et al.* Molecular understanding of sulphuric acid–amine particle nucleation in the atmosphere. *Nature* **502**, 359–363 (2013).

9. Carlsson, P. T. M. *et al.* Neutral Sulfuric Acid–Water Clustering Rates: Bridging the Gap between Molecular Simulation and Experiment. *J. Phys. Chem. Lett.* **11**, 4239–4244 (2020).

10. Myllys, N. *et al.* Role of base strength, cluster structure and charge in sulfuric-acid-driven particle formation. *Atmospheric Chem. Phys.* **19**, 9753–9768 (2019).

11. Kürten, A. *et al.* Experimental particle formation rates spanning tropospheric sulfuric acid and ammonia abundances, ion production rates, and temperatures. *J. Geophys. Res. Atmospheres* **121**, 12,377-12,400 (2016).

12. Stein, A. F. *et al.* NOAA's HYSPLIT Atmospheric Transport and Dispersion Modeling System. *Bull. Am. Meteorol. Soc.* **96**, 2059–2077 (2015).

13. Bergman, T. *et al.* Geographical and diurnal features of amine-enhanced boundary layer nucleation. *J. Geophys. Res. Atmospheres* **120**, 9606–9624 (2015).

14. Julin, J. *et al.* Impacts of Future European Emission Reductions on Aerosol Particle Number Concentrations Accounting for Effects of Ammonia, Amines, and Organic Species. *Environ. Sci. Technol.* **52**, 692–700 (2018).
